# Leveraging transcriptome and epigenome landscapes to infer regulatory networks during the onset of sexual maturation

**DOI:** 10.1186/s12864-022-08514-8

**Published:** 2022-06-01

**Authors:** Amin R. Mohamed, Marina Naval-Sanchez, Moira Menzies, Bradley Evans, Harry King, Antonio Reverter, James W. Kijas

**Affiliations:** 1 CSIRO Agriculture and Food, Queensland Bioscience Precinct, St Lucia, Brisbane, QLD 4067 Australia; 2 Present Address: Zoology Department, Faculty of Science, Benha University, Benha, Egypt; 3 Present Address: Institute of Molecular Bioscience (IMB), The University of Queensland, St Lucia, Brisbane, QLD 4067 Australia; 4 Tassal Operations Pty Ltd, Hobart, TAS 7001 Australia; 5 CSIRO Agriculture and Food, Hobart, TAS 7004 Australia

**Keywords:** Transcriptome, Epigenome, Puberty, Networks, Multiomics

## Abstract

**Background:**

Despite sexual development being ubiquitous to vertebrates, the molecular mechanisms underpinning this fundamental transition remain largely undocumented in many organisms. We designed a time course experiment that successfully sampled the period when Atlantic salmon commence their trajectory towards sexual maturation.

**Results:**

Through deep RNA sequencing, we discovered key genes and pathways associated with maturation in the pituitary-ovarian axis. Analyzing DNA methylomes revealed a bias towards hypermethylation in ovary that implicated maturation-related genes. Co-analysis of DNA methylome and gene expression changes revealed chromatin remodeling genes and key transcription factors were both significantly hypermethylated and upregulated in the ovary during the onset of maturation. We also observed changes in chromatin state landscapes that were strongly correlated with fundamental remodeling of gene expression in liver. Finally, a multiomic integrated analysis revealed regulatory networks and identified hub genes including *TRIM25* gene (encoding the estrogen-responsive finger protein) as a putative key regulator in the pituitary that underwent a 60-fold change in connectivity during the transition to maturation.

**Conclusion:**

The study successfully documented transcriptome and epigenome changes that involved key genes and pathways acting in the pituitary – ovarian axis. Using a Systems Biology approach, we identified hub genes and their associated networks deemed crucial for onset of maturation. The results provide a comprehensive view of the spatiotemporal changes involved in a complex trait and opens the door to future efforts aiming to manipulate puberty in an economically important aquaculture species.

**Supplementary Information:**

The online version contains supplementary material available at 10.1186/s12864-022-08514-8.

## Introduction

Epigenetic regulation of gene expression influences a vast spectrum of complex traits, with examples spanning the onset and severity of human disease, developmental transitions during growth and the expression of ecologically and economically relevant traits across the animal kingdom. Our understanding of the epigenetic contributions to trait variation remains low in comparison to causative genes derived from approaches such as genome wide association studies (GWAS). However, the development of sophisticated sequence-based assays for the detection of chromatin state changes and methylation status have enabled the landmark development of genome wide maps of regulatory elements in human [1–3], mouse [4–6] and other model organisms such as the fruit fly (*Drosophila melanogaster*) and the nematode (*Caenorhabditis elegans*) [7, 8]. These have provided the impetus for a plethora of research focused on understanding epigenetic mechanisms and their role regulating gene expression.

Sexual maturation is a fundamental transition ubiquitous to vertebrates and provides a model for the study of epigenetic regulation. The key tissues are known given the reproductive cycle is regulated by activation across the brain, pituitary, gonadal (BPG) axis in organisms spanning from mammals to teleost fish. Further, upon maturation these tissues undergo known and often profound transcriptomic remodeling providing a large dynamic range to increase the likelihood of identifying regulatory networks [9–12]. An energy-intensive process such as sexual maturation involves the liver as energy homeostasis is linked to fertility (Mircea et al. [13]; Montagner et al. [14]). Additionally, several regulatory transcription factors [11] and single-nucleotide polymorphisms were identified in the liver Insulin-like growth factor 1 (IGF1) gene to be associated with age at puberty in cattle (Fortes et al. [15]).

The genetic architecture of sexual maturation has been extensively studied using association studies in both wild and farmed Atlantic salmon populations [16–19]. However, a single omic dataset would not be appropriate to unravel the mechanisms underlying a complex physiological transition such as sexual maturation. Indeed, several multiomics studies in livestock species showed the promise of Systems Biology approaches to integrate multiple omics layers from different tissues to understand the molecular basis of complex traits including sexual maturation, feed efficiency and host-parasite interactions (Cánovas et al. [10]; Alexandre et al. [20]; Gòdia et al. [21]; Botwright et al. [22]).

Despite its importance as a trait of interest, sexual maturation can be difficult to study, as the timing of onset varies widely in response to both genetics and environmental factors and occurs prior to measurable phenotypic change. To overcome this, we chose to investigate sexual maturation in Atlantic salmon where photoperiod manipulation in an experimental system can be used to synchronize animals and access tissues across the time period when animals first commit to the onset of puberty. We also chose a multiomics approach, which has the power to identify the control mechanisms underpinning complex traits [23, 24]. We describe changes in gene expression, DNA methylation and chromatin accessibility to study transcriptional and epigenomic remodeling before inferring regulatory networks associated with onset of maturation in a commercially important aquaculture species.

## Results

### Initiation of salmon sexual maturation and a multiomic workflow

**Fig. 1:**
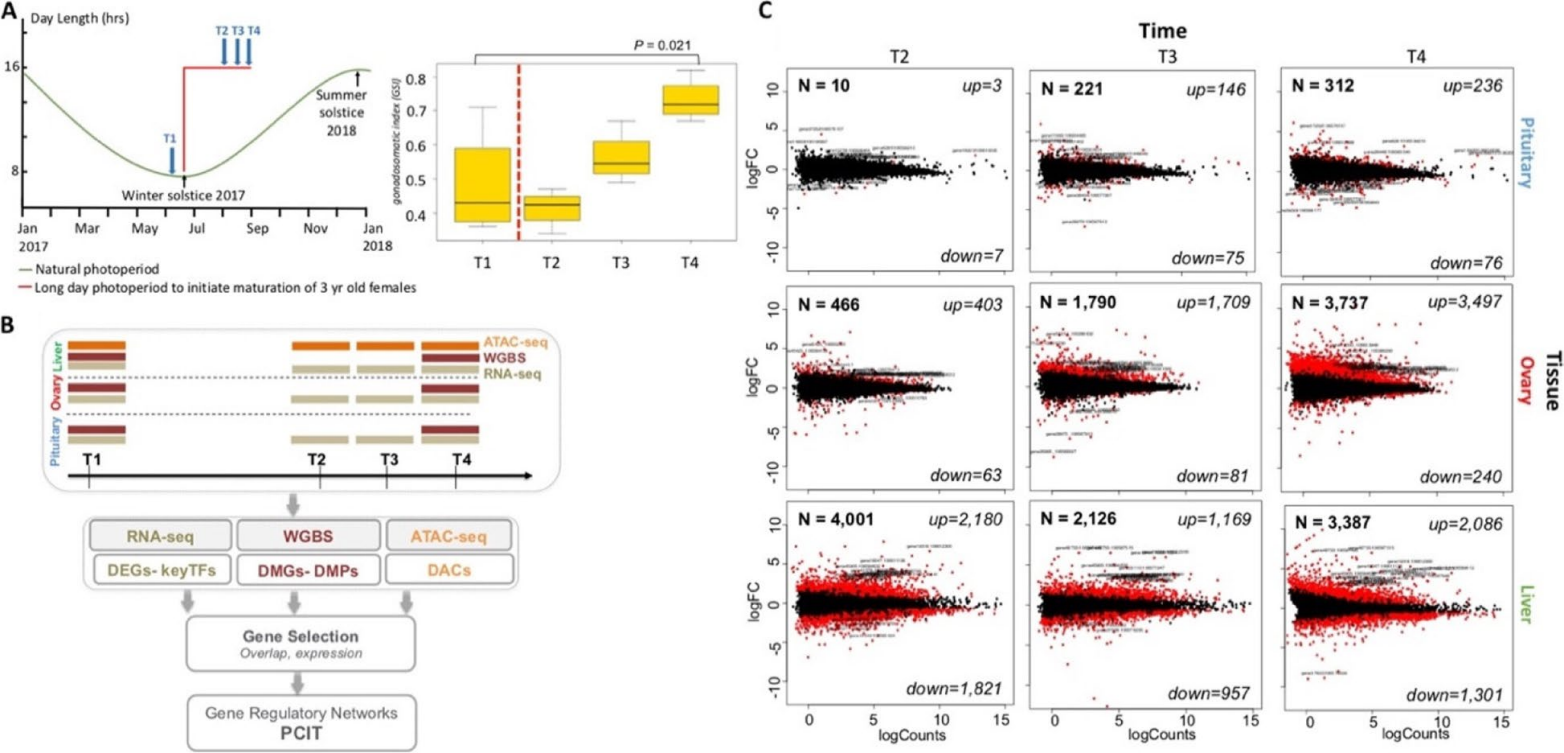
Transcriptomic and epigenomic changes associated with onset of salmon maturation (**A**), Induction of maturation through photoperiod manipulation and sampling time points. Animals were managed via photoperiod to synchronise the timing of commitment into maturation. 4 fish were sampled at each of the T1 (before long day photoperiod signal) and T2-T4 time points (during maturation) in 2 weeks intervals to control for variation between individuals. Gonadosomatic index (GSI) increased gradually from T2 till the last sampling event at T4 indicating active response towards maturation in these animals (**B**), Tissue collection and multi-omics analyses. Samples from the pituitary gland, ovary and liver were collected at each sampling event. Transcriptome and DNA methylome data in the three tissues along with accessible chromatin data in liver were collected (**C**), Significant transcriptome remodelling driving salmon maturation. 9 MA plots (showing differentially expressed genes (DEGs) in pituitary, ovary and liver (FDR < 0.05 and log_2_fold change > ±1) at T2, T3 and T4 during maturation compared to control samples at T1

Female fish were managed in a tank-based experimental system to facilitate a long-light photoperiod regime known to stimulate the onset of sexual maturation (Fig. 1A) [25, 26]. Fish from a single management group were sacrificed at a timepoint immediately before initiation of the long-light regime (throughout referred to as T1) and at three timepoints afterwards (T2, T3 and T4). An increase in gonadal somatic index (GSI) of sampled fish across the time course confirmed an active response to the long photoperiod (Fig. 1B). Significant increases were observed only at T4 (t-test *P*-value = 0.021). Tissues from the BPG (brain – pituitary – gonad) axis and liver were sampled at each timepoint to form the basis of a multiomics workflow for data generation and integrative analysis spanning the transcriptome, DNA methylome and chromatin state datatypes (Fig. 1B; Supplementary Fig. S1).

### Significant transcriptome changes in the pituitary-gonad axis reveal candidate genes and regulators of maturation onset

We sequenced messenger RNA (mRNA) from four biological replicates of each tissue (brain, pituitary, ovary and liver) before and after the onset of maturation. A total of 3.2 billion paired-end reads were mapped against the Atlantic salmon reference genome with 72% mapping efficiency to create an average depth of 50 million reads per library (Data S1). Consistency across biological replicates within each timepoint was high for each tissue except for brain. Brain samples were excluded from further analyses because of inconsistency among replicates (see Supplementary Results; Supplementary Fig. S2).

To begin characterization of transcriptomic remodeling, we compared gene expression in three post-maturation samples (T2, T3 and T4) against the control T1 in three tissues (pituitary, ovary and liver) (Fig. 1C). The pituitary gland showed comparatively subtle transcriptomic responses that involved 543 differential expressed genes (DEGs; adjusted *P* < 0.05 and log2FC > ±1) (Data S2). In contrast, more widespread remodeling was observed in both ovary (5993 DEGs) and liver (9541 DEGs) (Fig. 1C, Data S2) using the same significance and fold change thresholds. The number of DEGs increased with elapsed time following the onset of the long light photoperiod for the two BPG axis tissues (pituitary and ovary). Of these, the ovary underwent the most dramatic remodeling over time with 403, 1709 and then 3497 DEGs observed at timepoints T2, T3 and T4 respectively (Data S2). This increasing trajectory of differential gene expression in ovary, coupled with the elevated GSI following the light stimuli (Fig. 1A), strongly suggests the experimental approach successfully initiated the onset of maturation.

**Fig. 2:**
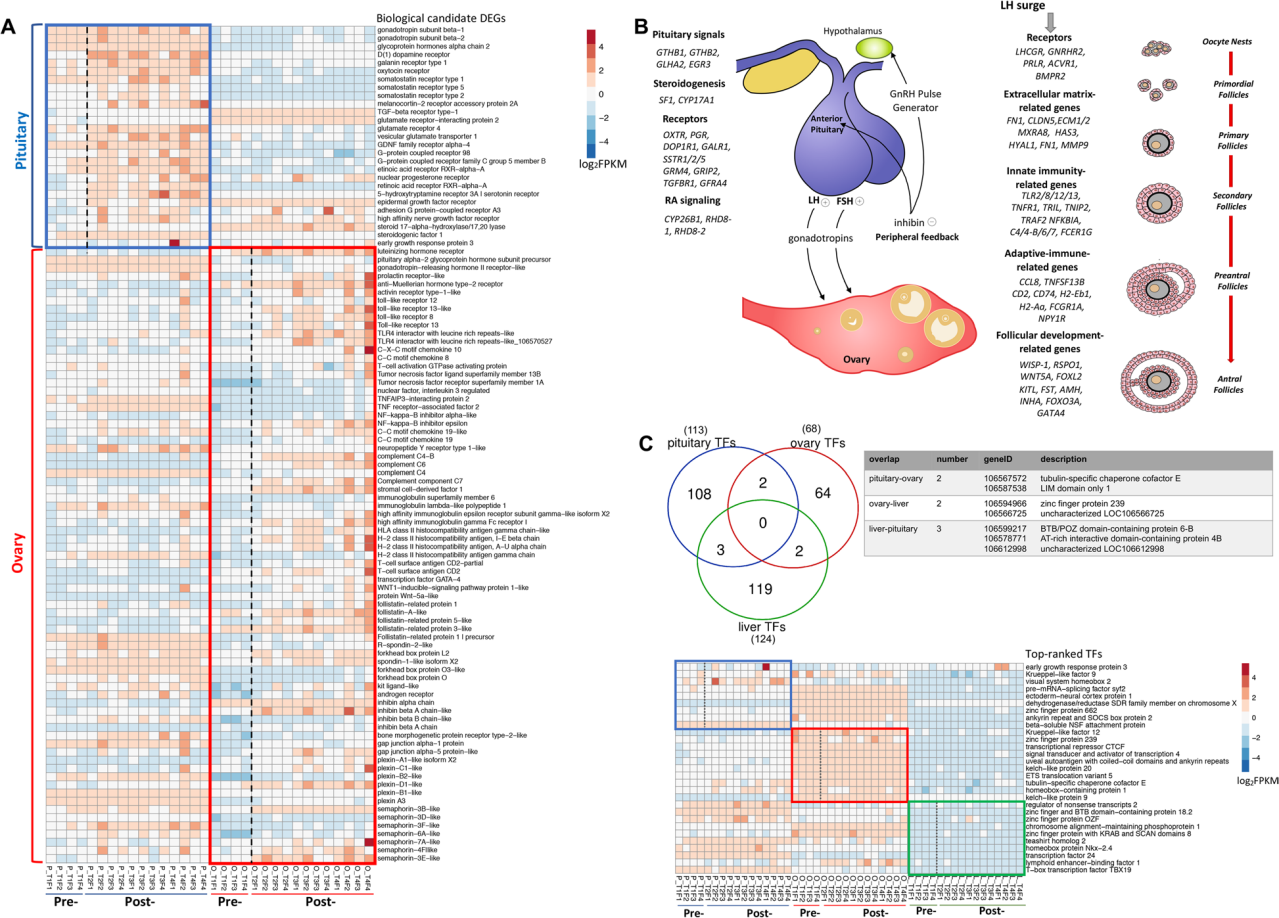
Activation of maturation candidate genes and regulatory factors in the pituitary-gonad axis (**A**), Heat map of key biological candidates with significant expression changes in pituitary and ovary during onset of maturation. The clustering shown was obtained by comparing the normalised expression values (log_2_-transformed and mean-centered FPKM values). The red-blue scale represents the relative expression values (**B**), Model of genes implicated in activation of the pituitary-ovarian axis in Atlantic salmon and their potential roles during maturation onset. Panel **B** was adapted from [32, 33] (**C**), Key transcription factors (TFs) with regulatory potential identified based on co-expression with DEGs at T4 using RIF metrics. The Venn diagram shows overlap of these TFs among the three tissues. The heatmap shows normalized expression of top10 factors per tissue ranked based on their RIF scores

The pituitary is expected to play a key role in the early stages for maturation onset [27]. Hierarchical clustering of pituitary DEGs revealed two distinct gene clusters (via hierarchical clustering), one of upregulated genes (*n* = 333; 60% of pituitary DEGs) and one of downregulated genes (*n* = 125). The upregulated cluster showed significant gene ontology (GO) enrichment for maturation-related functions including G protein−coupled receptor signaling and hormone activity (Supplementary Fig. S4 and Data S3). Pituitary DEGs were involved in several reproduction-related functions such as steroidogenesis (*SF1, CYP17A1*), hormone receptors (*GRM4, OXTR, PGR, DOP1R1, GALR1*), genes coding for gonadotropins (*GTHB1, GTHB2, GLHA2*) and retinoic acid (RA) signaling (*CYP26B1, RHD8*–1/2) (Fig. 2A). The upregulation of gonadotropins subunits is highly significant in the context of onset of puberty. This included both *GTHB1* and *GTHB2* that encode the gonadotropin subunits beta-1 and 2 as well as *GLHA2* that encodes the glycoprotein hormone alpha chain. Together, these form the heterodimeric gonadotropins (GTH-1 and GTH-2) that have previously been shown to stimulate gonadal growth in the juvenile stages of both rainbow trout and coho/chum salmon [28–30]. Further, physicochemical characterization of the salmon gonadotropins indicate they are functionally related to follicle stimulating hormone (FSH) and luteinizing hormone (LH) in vertebrates (reviewed by [31]). We find *GLHA2* gene (the common subunit present in gonadotropins) was consistently upregulated in the pituitary throughout the experiment (Supplementary Fig. S3). Clustering DEGs in ovary revealed distinctive expression profiles for post-maturation and two distinct clusters of upregulated genes (*n* = 3476; 58% of ovary DEGs) and downregulated genes (*n* = 301) were identified (Supplementary Figs. 3 and 5A, B). The functional profile for upregulated genes in the ovary revealed processes related to cell adhesion, immune response and regulation of development (Supplementary Fig. S5; Data S3). Examination of ovary DEGs identified several gene categories related to follicular development. Following LH surge, several hormonal receptors (including *LHR, GNRHR2, and AMHR2*) were upregulated leading to activation of extracellular matrix proteins such as *CLDN5, ECM1/2, MXRA8*, and *HAS3*. The maturation response was also associated with upregulation of immune-related genes such as *MYD88T, TLR, CD2, IGLL1*, *CCL19*, and *TRAF2*. Key transcription factors (TFs) controlling ovarian development (*FOXL2* and *GATA4*) along with follicular developmental genes (*RSPO1, KITL, FST, AMH, FSTL1* and several sema/plexin genes) and steroidogenesis genes (*CYP17A1, SF-1* and *STAR*) were also upregulated (Fig. 1A; Supplementary Fig. S6).

In liver, many genes were differentially expressed at all end time points (T4) (Data S4). Hierarchical clustering of the liver DEGs revealed distinctive expression profiles for post-maturation and identified two distinct clusters of upregulated genes (*n* = 3336) and downregulated genes (*n* = 2347) (Supplementary Fig. S7). Upregulated gene cluster showed significant GO enrichment with respect to 45 GO-BP, 17 GO-CC and 23 GO-MF terms related to organic acid metabolic processes and mitochondrial transport (Supplementary Fig. 7 and Data S3), consistent with the understood role the liver plays to provide the energetic potential required for the transition to puberty. Taken together, the multi-tissue transcriptomic profiling identified key genes in pituitary as early triggers and ovarian genes as downstream effectors in ovary. A model of the pituitary-gonad axis genes and their roles during onset of sexual maturation is presented in Fig. 2B.

Next, we performed regulatory impact factor (RIF) analysis to identify key TFs significantly contributing to differential expression between endpoints T1 and T4 samples (where most of the transcriptomic changes have occurred). Predicted TFs (obtained from [34]) and their target DEGs at T4 were used to identify regulators with significant scores (deviating > ± 2.57 standard deviation from the mean; *P* < 0.01) (Fig. 2C; Data S4). This identified a total of 305 significant regulators (113, 68 and 123 in pituitary, ovary and liver). Most were tissue specific (*n* = 298; 97.7%) with only 7 shared among tissue pairs (Fig. 2C). Next, we ranked these regulators based on their absolute RIF1 (based on consistent differential co-expression with DEGs) and RIF2 (based on the most altered ability to predict the abundance of DEGs) scores to identify the top-10 factors and plotted their normalized expression to show higher expression in the pituitary-ovary axis (Fig. 2C). While the majority of these genes didn’t show significant changes in expression between the two time points, they were highly scored by RIF due to significant differences in correlation with DEGs in the two conditions (differential wiring). Four zinc finger proteins (such as *ZNF662* in pituitary and *ZNF239* in ovary) were among these top factors, suggesting that these members of the ZNF gene superfamily may be involved in regulating onset of maturation in salmon. In fact, the potential contribution of ZNF genes to the maturation process has been previously shown [35]. Among the pituitary top regulators, *ERG-3* encoding early growth protein 3 was highly expressed in the post-maturation samples compared to the control. ERG-3 belongs to a family of immediate early response genes that contains a conserved zinc finger DNA-binding domain that binds to GC-rich sequence in the promoter regions of target genes [36]. A member of the early growth protein family, ERG-1 along with nuclear receptor steroidogenic factor 1 (SF-1) (differentially expressed in pituitary transcriptome data) are essential TFs required for LHβ gene expression in the pituitary where EGR-1 binds and activates the LHβ promoter in combination with SF-1 [37].The DNA binding domain of EGR-1 is highly homologous among other members of this family, including EGR-3 [38].The expression patterns of both *ERG-3* and *SF1* suggests similar functions in female reproductive development in Atlantic salmon. Taken together, we showed that key regulators were identified based on their co-expression with DEGs, leading to a deepened understanding of gene regulation during maturation.

### Genome wide increase in CpG methylation levels in ovary during maturation

To investigate the regulatory mechanisms controlling differential gene expression, we constructed genome-wide CpG methylation maps for Atlantic salmon using whole-genome bisulfite sequencing (WGBS). Methylome data was collected from two biological replicates at the terminal time points (T1 and T4, Fig. 1B) from the three tissues (pituitary, ovary and liver), generating 2.6 billion paired-end uniquely mapped reads (average coverage of 11x) (Data S4). We found an average genome-wide methylation rate of 81% per sample (Supplementary Fig. S8; Data S5), similar to the rate observed in vertebrate genomes (60–90%) [39, 40]. Methylome data was assessed based on coverage, read mapping and consistency between biological replicates (see Supplementary Results; Data S5; Supplementary Fig. S8). Among the different dinucleotide contexts, CpG methylation contributed the vast majority (~ 99.5% on average) compared to CHH or CHG methylation which were excluded from further analysis (Supplementary Fig. S8; Data S5).

**Fig. 3:**
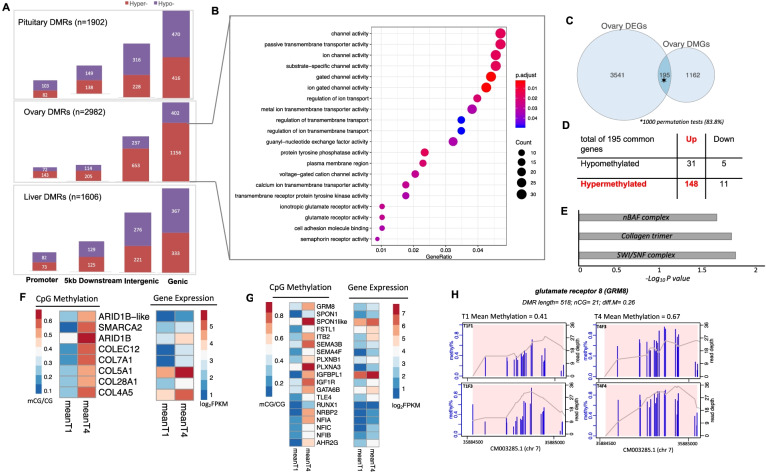
Multi-tissue DNA methylome maps reveal differentially methylated genes with key roles in salmon maturation (**A**), Changes in CpG methylation across four genomic regions per tissue, promoters were defined as regions located 5 kb upstream of transcription start sites (TSSs) (**B**), Significantly enriched gene ontology (GO) terms among ovary hypermethylated genes (*n* = 1156) against background of ovary genes (hypergeometric test, Bonferroni-adjusted *P* < 0.05) (**C**), Significant overlap between ovary DMGs and DEGs (**D**), Methylation and expression directionality at gene bodies confirms that gene body methylation is associated with gene activation (**E**), Significantly enriched GO terms (adjusted *P* < 0.05) among the list of 148 hypermethylated genes in ovary (**F**), Heatmaps showing significant increases in CpG methylation and expression of genes driving GO enrichment shown in part **F**. **G** Heatmaps showing significant increases in CpG methylation and expression of transcription factors and maturation candidate genes and among the list of the 148 genes. **H** CpG methylation profiles for the *GRM8* gene. The methylation plot shows percentages of methylated CpGs along with coverage depth at each CpG site. Pink rectangles represent the DMR and genomic coordinates are indicated below the density plot

By comparing CpG methylation patterns between T4 and T1 timepoints, we identified 1902, 2982 and 1606 differentially methylated regions (DMRs) in the pituitary gland, ovary and liver respectively (Supplementary Fig. 9A, B; Data S6). The average length of DMRs was short (251 bp) and their distribution was both genome wide (Supplementary Fig. 9C) and strongly tissue specific, with few regions shared between tissue pairs and only 8 found in all three tissues (Supplementary Fig. 9D). The genomic location of these DMRs were highly non-random, with 52% found to overlap protein coding genes and another 18% located within 5 kb upstream or downstream of coding genes (Supplementary Fig. 9E). Next, we investigated the directionality of DMRs across tissues and found approximately equal rates of hyper-methylation (increased methylation in T4) and hypo-methylation (decreased methylation in T4) in both the pituitary and liver (Fig. 3A). Strikingly, the majority of DMRs in ovary were hyper-methylated (2175 DMRs or 73%). The increase in methylation occurred independent of genomics location (Fig. 3A), however we identified many fewer DMRs in promoters (7% of the 2175 DMRs) and 5 kb downstream of genes (11%) compared with those located in coding regions (52%) or within intergenic regions (30%). Given the ovary underwent the largest increase in upregulation of gene expression and had the highest number of DMRs, hyper-methylation appears to have played a role. This is consistent with DNA methylation having important roles during epigenomic reprograming in embryo and stem cell development [41], compared to highly stable methylomes in somatic cells [42]. Further, increased methylation response has been associated with the initiation of human puberty [43] and the ovary also undergoes the most radical physiological change during maturation as it transforms via vitellogenesis and oocyte development in preparation for egg release during spawning. The gene catalogue present within differentially methylated regions (DMRs) was assessed for their function in relation to the trait. The majority of ovarian differentially methylated genes (DMGs) were hypermethylated (*n* = 1165; 74%) (Fig. 3A) and significantly enriched for three biological process (GO-BP), one cellular component (GO-CC) and 24 molecular function (GO-MF) terms including those with maturation-related functions such as semaphorin, glutamate receptor activity and cell adhesion molecule binding (Fig. 3B; Data S7). Several genes encoding Semaphorin and Plexin proteins were upregulated in ovary (Fig. 2A) implicating a role during ovary development. In mouse, Semaphorin-4D/Plexin-B1 binding is involved in ovary follicular development through extracellular matrix re-organization, cell adhesion and proliferation [44–46].

### Co-analysis of DNA methylomes and transcriptomes implicated chromatin remodeling genes and key transcription factors

To search for evidence of a generalized and genome-wide association between gene expression levels and methylation status, expression levels for all genes with either differential gene body or promoter methylation were evaluated (Supplementary Fig. S10A, B). This revealed no correlation for either comparison, consistent with previous studies reporting weak correlation between global changes in DNA methylation and gene expression in different species including humans [47, 48] and more recently in fish [49]. We next explored the dynamic between DNA methylation and gene expression by assessing the overlap of genes declared as both DEG and DMG. The overlap was low and non-significant for liver (38 / 616 or 6% of DMGs were also DEGs) and pituitary (11 / 762 or 1.4% of DMGs were DEGs) (Supplementary Fig. S10C). However, 195 or 14% of ovary DMGs (195/1357) were also DEGs, a number that exceeded random expectation in 83.8% of 1000 permutations tests (Fig. 3C). This suggests changes in methylation status may directly control gene expression in a targeted subset of genes. If true, we would expect to see correspondence between the directionality of the expression and methylation changes. This appeared to be the case, as 82% of upregulated genes (148 / 179; Binomial *P*-value = 8.727E^− 20^) were hyper-methylated at T4 relative to T1 (Fig. 3D), matching the conventional expectation of gene body methylation mediated control of gene expression [50, 51]. The 148 genes were enriched for 3 GO-CC terms related to chromatin remodelling complexes (SWI/SNF and nBAF) (Fig. 3E; Data S7), and the associated genes displaying coordinated expression and methylation status (Fig. 3F). For example, the ARID1B gene encodes AT-rich interactive domain-containing protein 1B and SMARCA2 encodes the global transcription activator SNF2L2. Both proteins are involved in chromatin remodeling as they are core components of the Switching defective/sucrose nonfermenting (SWI/SNF) remodeling complexes [52] that carry out enzymatic change to chromatin structure by altering DNA-histone contacts [53]. These complexes are evolutionary conserved protein complexes involved in regulating diverse pathways such as differentiation and cell proliferation including gynecologic cancer (Wang et al. [51]). Indeed, many components of the SWI/SNF complexes have been implicated in embryonic germline development (Klochendler-Yeivin et al. [52]) including initiation of sex-dependent differentiation in mouse (Ito et al. [53]).

Several TFs implicated in ovary development were also present among the 148 hyper-methylated and upregulated genes. Plotting the normalized mean expression and methylation suggests a coordinated response (Fig. 3G; Data S7). Prominent examples include *RUNX1* that is a critical TF for cell lineage specification [54] and ovarian development though interaction with *FOXL2* [55]. Another gene, *TLE4*, encodes transducin-like enhancer of split 4 that is a member of the TLE co-repressor superfamily, some of which are associated with female fertility [56]. Finally, AHR2G encodes aryl hydrocarbon receptor 2 gamma and is implicated in regulating growth of ovarian follicles in mice [57]. This raises the possibility these regulatory genes serve as key regulatory factors, acting to regulate a wide array of other genes with important functions during ovarian development. Prominent candidates are IGF-1 encodes insulin like growth factor 1 that stimulates either proliferation, or differentiation of granulosa cells [58] following FSH secretion. Other genes involved in follicular development (*SPON1, PLEXNB1, SEMA4F*) [41–43] and the control of gonadotropin-releasing hormone excitability via glutamate receptor 8 [59] (Fig. 3H). Taken together, the results confirmed that while methylation alone does not control genome-wide patterns of gene expression, our integrated methylome/transcriptome analysis demonstrated that coordinated response of core components of the SWI/SNF complex and specific TFs with genes involved in ovary development, suggesting key roles during the onset of the maturation process in the target organ (ovary).

### Differential chromatin accessibility strongly correlates with bidirectional regulation of global gene expression in liver

To deepen the characterization of the epigenomic features during maturation, we performed ATAC-seq (assay for transposase-accessible chromatin sequencing [60]) to produce genome-wide maps of chromatin accessibility changes. ATAC-seq was performed for ovary among the other tissues and peak enrichment around transcription start sites used as the key quality control metric (TSS, Supplementary Fig. S11). Following data pruning, we took only 12 liver libraries (3 replicates across all 4 timepoints) and a total of 699 million uniquely mapped paired-end reads (Data S8) forward into joint analysis with RNA-seq and WGBS data.

**Fig. 4:**
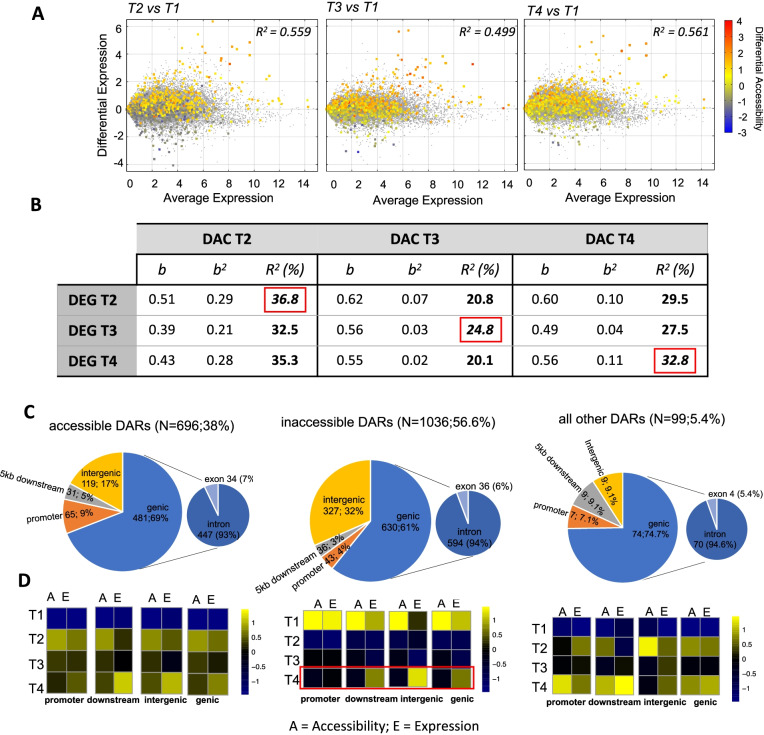
Dynamics of liver chromatin accessibility and gene expression as a function of genomic location (**A**), Chromatin accessibility and gene expression are positively correlated. 3 MA biplots showing genome-wide gene expression and overlain differentially accessible regions at gene bodies at T2, T3 and T4 compared to T1. Accessibility levels are shown in red-blue spectrum reflecting open- to closed chromatin states at gene bodies and the corresponding gene expression in grey colour (**B**), Regression analyses conducted on significant DARs located at gene bodies and gene expression data. The table shows that accessibility at T2 explains the majority of the observed differential expression throughout the experiment. Red squares highlight the correlation of the paired accessibility and expression data of the same time point (**C**), The genomic distribution of co-accessible DARs clusters across gene models in the salmon genome, promoter defined as genomic regions located 5 kb upstream of TSSs (**D**), Multi-omic heatmaps showing mean accessibility and expression at each of the four time points per genomic location

To characterize changes in chromatin state following long light initiation, we defined differentially accessible regions (DARs) where mapping counts differed significantly (at adjusted *P* < 0.05 and log_2_FC > ±1) between T1 and other time points (Data S8). The direction of change was approximately balanced between DARs with increased and decreased accessibility, broadly matching the balance between up and down regulated global gene expression changes observed for liver (Fig. 1C). Starting with the subset of DARs co-located with genes (exons and introns) we found the proportion of variation in gene expression explained by chromatin accessibility changes was high (Fig. 4A, B, Supplementary Fig. S12). For example, chromatin state changes at T2, compared with T1, explained 56% of the variation in gene expression using linear regression. The dynamic was bidirectional, with accessibility changes associated with both up and down regulation of global gene expression, and strongest at the early timepoint T2 (Fig. 4B). We repeated the analysis for DARs located within 5 kb of transcription start sites to assess the strength of association with physically proximal putative cis-regulatory elements (CREs; non-coding genomic regions that could regulate expression of nearby genes). These had even higher association, explaining approximately 60% of the variation in global gene expression (Supplementary Fig. S13).

Hierarchical clustering of the significant DARs revealed 3 distinct clusters of similar accessibility patterns (Supplementary Fig. 14; Data S8). To explore the relationship between chromatin accessibility changes and gene expression, we first mapped the genomic location of DARs and found the majority were located in genes (65%) or within 5 kb upstream (6%) or 5 kb downstream (4%), affirming the quality of the ATAC-seq dataset (Fig. 4C). We also detected a quarter of DARs located on average 36 kb distal to their nearest gene, a low proportion in comparison to domesticated terrestrial livestock [61]. Next, we plotted the mean normalized accessibility and expression across the four genomic locations to show that CREs more tightly controlled the downregulation of genes compared to DARs located in gene bodies, downstream regions or within intergenic regions (Fig. 4D, red box). Next, we focused on CREs given their established role on transcriptional regulation via TF binding [62]. Intersecting the co-accessible cluster of DARs with gene models in the salmon genome (Supplementary Fig. S14), identified a small subset of CREs (*n* = 65) underwent increased accessibility early in the time course (Fig. 4C) and the majority (*n* = 46; 79%, Χ^2^*P* < 8.028^− 08^) of their nearest genes were upregulated in a tightly coordinated manner (Supplementary Fig. S15). The gene set associated with coordinated up regulation exhibited significant GO enrichment related to lipid metabolism and energy metabolism (AAcyl-CoA biosynthesis) (Supplementary Fig. S15). Acyl-CoA are coenzymes involved in energy synthesis, consistent with the expectation of liver function through an energetically costly transition such as maturation. Together, the previous results clearly demonstrated chromatin state changes played a dominant role in directing global gene expression changes in liver.

### Integration of multiomics data to infer gene regulatory networks underlying maturation onset

**Fig. 5:**
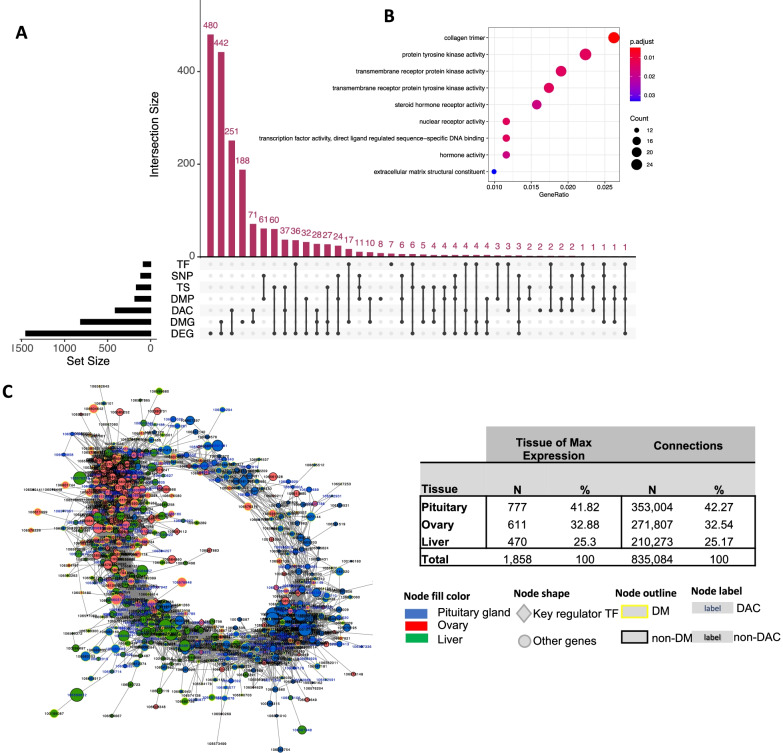
Multi-omics derived gene regulatory network (**A**), Intersection among the 1858 genes selected for network analyses. Network genes were divided into 7 categories: differentially expressed genes (DEG), differentially expressed genes/promoters (DMG, DMP), differentially accessible regions (genic and promoter regions as DAC), genes harboring SNPs reported in literature as being associated with salmon maturation (SNP), tissue-specific genes (TS) and key regulators identified by RIF (TF). The nature of a given intersection is indicated by the dots below the bar plot. For example, the 442 genes in the second column are both differentially methylated and differentially expressed but not found in other categories (**B**), Enriched gene ontology (GO) terms (hypergeometric test, Bonferroni-adjusted *P* < 0.05) among the list of 1858 network genes (**C**), Gene regulatory network constructed using the PCIT algorithm, for visualization only nodes with significant correlations ≥ ±0.95 (929 genes with 17,708 connections) were considered. All nodes are represented by ellipses except for genes coding key regulators (TFs) have diamond shape. Nodes with yellow borders are differentially methylated, whereas nodes with white labels are differentially accessible. Node colours are relative to the tissue of maximum expression with blue represents the pituitary, red represents ovary and green represents liver. The size of the nodes is relative to the normalized mean expression values in all samples. Connectivity structures of the 1858 network genes used to build the network are shown

The final component of our analysis sought to integrate all available datatypes to infer gene regulatory networks (GRNs) responsible for the onset of maturation. GRNs provide a platform for integrating multiomic data and can be used to characterize the dynamics of perturbations during biological transitions such as embryogenesis, puberty, and other complex physiological evolutions [63–67]. Here, we used the approach to co-analyze genes with evidence of differential behavior using seven categories that included expression (DEGs and key TFs), changed methylation at gene bodies (DMGs) or promotors (DMPs) and differential chromatin accessibility (DACs) at genes and putative promoters (defined as regions 5 kb upstream of a transcription start site TSS). Of the seven categories, the majority of 1858 genes prioritized for GRN construction were DEG (*n* = 1400) or DMG (*n* = 700). The overlap between categories, for example where genes were both DEG and DMG (*n* = 442), is given in Fig. 5A. The gene set showed significant GO enrichment (1 GO-CC and 8 GO-MF terms) to hormone activity and steroid hormone receptor activity (Fig. 5B). The expression patterns of these genes not only showed clear tissue-specific clustering, but also resolved the maturation status of the samples (Supplementary Fig. S17) suggesting biological relevance to the trait under investigation.

GRN inference using 1858 genes yielded 835,084 connections with a mean of 449 connections per gene. For visualization, we only considered gene-gene connections with significance according to the PCIT algorithms and from correlations ≥0.95 (929 gene with 17,708 connections) (Fig. 5C). Most network genes (*N* = 777, ~ 42%) belonged to pituitary compared to 33 and 25% in ovary and liver. These figures also were reflected in the number of connections per tissue (Fig. 5C). Genes with the highest change in the number of connections are likely to be key regulators, and the top 20 included five zinc finger proteins (Data S9). Two of these transcription factors, ZNF664 and ZNF239, were expressed in pituitary suggesting their key role in maturation onset. Interestingly, the most highly connected genes also included two uncharacterized (dark) Atlantic salmon genes (106,590,493, 106,612,553) that displayed maximum expression in ovary.

### Differential GRN connectivity implicates TRIM25 and its associated subnetwork as key mediators of the transcriptional responses

**Fig. 6:**
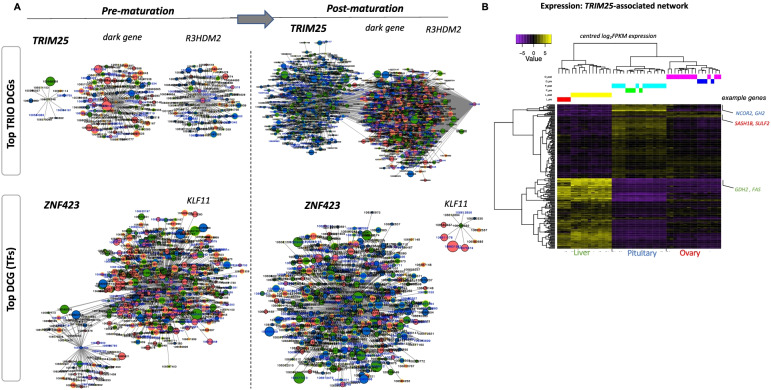
Pre- and Post-maturation networks based on differentially connected genes (DCGs) (**A**), Subnetworks of the most differentially connected trio genes between pre- and post-maturation networks (top). This revealed *TRIM25*, a E3 Ubiquitin ligase as the key regulator with the greatest number of gained connections in the post-maturation network. Subnetworks of top differentially connected regulators (TFs) (bottom), networks created with the most differentially connected TFs between pre- and post-maturation networks showed zinc finger protein 423 (*ZNF423*) as the key regulator with the greatest number of gained connections and Kruppel-like factor 11 (*KLF11*) as the regulator with the least number of gained connections going from pre- to post-maturation (**B**), Expression patterns of genes of the *TRIM25*-asscoiated network. The heatmap shows hierarchical clustering based on mean-centred normalised (log_2_FPKM) expression values

Key regulators are likely to undergo substantial change in their number of connections and identify gene networks driving the transition to maturation. This prompted construction of separate networks using pre- and post-maturation stage data, before identifying those “hub” genes that underwent the largest change in connectivity (pipeline workflow is provided in Supplementary Fig. S1B). For visualization, we only included 10% of the most significant connections that included 1412 genes with 17,260 connections in the pre-maturation GRN and 1310 genes with 22,059 connections in the post-maturation GRN (Supplementary Fig. S18). Next, we computed the differences in the patterns among the tissues comprising the two networks. The pituitary gland and ovary had the most abundance (~ 45 and 32%, respectively) of connections compared to a lower percentage of connections (~ 23%) in liver after maturation (Supplementary Fig. S18). We computed the differential connectivity for all genes and identified the most differentially connected genes (DCGs) (*n* = 186 genes; 10%) (Data S9) between the pre- and post-maturation networks. These were mainly expressed in pituitary (44%) and most connections involved DEGs (74%) and DMGs (47%). Finally, we identified regulators that gained the most connections post-maturation (Table 1, Fig. 6). The top ranked regulators were *TRIM25*, *R3HDM2* and a salmon dark gene (uncharacterized 106,590,493) that was differentially methylated and highly expressed in ovary (Fig. 6A). TRIM25 encodes the estrogen-responsive finger protein (a ubiquitin E3 ligase) underwent a profound change (60-fold) in connectivity (from 10 to 599). It is a ZF-TF with multiple roles in signal transduction during development. It belongs to the tripartite motif-containing (TRIM) family, most of which have E3 ubiquitin ligase activities implicated in innate immunity and tumorigenesis with multiple roles in signal transduction during development [68–70]. It was predominantly expressed in pituitary and contributed to 3 categories in the network (DEG, TF, DAC).

**Table 1:** Top 20 differentially connected genes (DCGs) associated with onset of sexual maturation in Atlantic salmon. Gene annotation, tissue of maximum expression, the omic category and differential connectivity data between pre- and post-maturation are shown

GeneID	Number of connections		Differential connectivity	Annotation	Contribution	Tissue of maximum expression	Omic category
	Pre-	Post-	Post vs Pre				
106,574,348	10	599	589	E3 ubiquitin/ISG15 ligase TRIM25-like	28.57	pit	DE, DMG
106,590,493	114	657	543	uncharacterized protein LOC106590493, partial	28.57	ov	DE, DMG
106,566,126	90	609	519	R3H domain-containing protein 2-like	28.57	ov	DE, DAC
106,573,705	58	576	518	zinc finger protein 423	42.86	pit	DE, TF, DAC
106,589,399	43	551	508	protocadherin-15-like	14.29	pit	DMG
106,562,515	4	508	504	RILP-like protein 1	28.57	liv	DE, DMG
106,562,229	30	533	503	tumor necrosis factor receptor type 1-associated DEATH domain protein-like	28.57	ov	DMG, DAC
106,612,386	41	535	494	zinc transporter protein DDB_G0282067	28.57	ov	DE, DMG
106,568,904	37	525	488	putative ferric-chelate reductase 1	14.29	ov	DE
106,568,722	10	489	479	cAMP-specific 3\’,5\’-cyclic phosphodiesterase 4B-like	14.29	pit	DMG
106,606,431	487	32	− 455	metalloproteinase inhibitor 2-like	14.29	pit	DE
100,195,217	543	87	− 456	protein phosphatase 1H	28.57	liv	DE, DAC
106,613,686	560	104	−456	Krueppel-like factor 2	28.57	liv	DE, DAC
100,195,880	474	11	− 463	Ependymin-1 precursor	14.29	pit	DE
100,195,838	488	18	− 470	ependymin-2 precursor	28.57	ov	DE, DMP
106,588,682	594	113	− 481	zona pellucida sperm-binding protein 3-like	14.29	pit	DE
106,610,009	546	46	−500	guanylate cyclase soluble subunit beta-1	28.57	liv	DMP, DMG
100,194,692	513	9	−504	Krueppel-like factor 11	28.57	liv	DE, TF
100,196,465	560	31	− 529	glyoxalase domain-containing protein 5	14.29	ov	DE
106,586,502	559	7	− 552	serine/arginine repetitive matrix protein 2-like	42.86	ov	DE, DMG, DAC

Next, we focused on TFs contained among the top 10 regulators that were differentially connected. This identified *ZNF423* to be the most differentially connected TF (from 58 to 576) and Krueppel-like factor 11 (*KLF11*) as the least differentially connected TF (from 513 to 9) (Fig. 6; Table 1). Enrichment of motifs for a ubiquitin E3 ligase (CNOT4) and ZNF TF (Zic) among ATAC-seq signals at CREs confirms their regulatory roles. Several studies have previously demonstrated roles for ZNF factors in controlling onset of female puberty in many species including humans [71–73]. Finally, we focused on the subnetwork associated with TRIM25 (being the top ranked regulator), the TRIM25-associated networks consisted of 213 genes (Supplementary Fig. S19) of which 108, 90 and 15 were highly expressed in liver, pituitary and ovary, respectively (Fig. 6B). We ranked the TRIM25-connected genes based on mean expression per tissue and identified key mediators of the transcriptional profiles observed in each tissue. NCOR2 encoding Nuclear Receptor Corepressor 2, a co-repressor that mediates transcriptional silencing of target genes and GH2 encoding the growth hormone somatotropin-2 were highly expressed in the pituitary. Genes involved in immune responses and cell signaling (SASH1B encoding SAM and SH3 domain-containing protein 1-like; involved in the TLR4 signaling pathway that may stimulate cytokine production/endothelial cell migration in response to pathogens and SULF2 encoding Sulfatase 2) were highly expressed in ovary. In liver, genes implicated in energy homeostasis (GDH2 encoding glutamate dehydrogenase that controls body energy partitioning through amino acid-derived gluconeogenesis and FAS encoding fatty acid synthase) were highly expressed (Fig. 6B).

## Discussion

Identifying the biological mechanisms controlling complex traits is a sizable challenge. We designed our study on the assumption that the dynamic network of molecules coordinating the spatiotemporal changes driving sexual maturation would be inaccessible to investigation using only a single layer of “omics”. We anchored the study around the collection of tissue transcriptomes to visualize their changing circuitry across the period where Atlantic salmon commence their trajectory towards sexual maturation. Importantly, we also characterized the changing epigenomic landscape through interrogation of DNA methylation and chromatin state changes. Integration of the resulting multiomic dataset used rigorous quantitative approaches, and when performed inside the context of a defined biological transition, has given us an unprecedented ability to characterize the onset of maturation at the molecular level in a non-model species of worldwide aquaculture and ecological importance.

The availability of dimensional data allowed us to identify the dominant epigenomic changes controlling gene expression. We conclude that global changes in DNA methylation had little predictive power to explain changing gene expression beyond a small subset involved in chromatin remodeling. While methylation changes are a striking feature of embryonic development, they appear not to have been responsible for the rapid and numerous changes in gene expression documented here. Conversely, we observed high correlation between chromatin state changes and altered gene expression for the single tissue with ATAC-seq data (liver). The correlation was highest for differentially accessible regions immediately adjacent to coding genes, implicating cis-regulatory elements.

The identification of key genes relied on characterization of differential behavior using samples collected before, and after, fish were subjected to a photomanipulation trigger designed to stimulate maturation. Multiple data patterns confirm we successfully initiated early-stage maturation. Increasing average gonadosomatic index demonstrates a physiological response occurred, and this was paralleled by significant global upregulation of gene expression in the ovary and a more modest remodeling of the pituitary transcriptome. Together, this provided confidence that the characterized DEG, DMG and DAC patterns are likely to successfully implicate genes directly involved in maturation. We showed upregulation of pituitary hormones including gonadotropins along with other pituitary genes involved in a range of reproduction related functions including steroidogenesis. Differentially methylated genes were enriched for follicular development and the control of gonadotropin-releasing hormone excitability. Integrated transcriptome and methylome analysis in ovary implicated chromatin remodeling genes in controlling maturation. Finally, differentially accessible CREs in liver were enriched for lipid metabolism and energy metabolism genes.

Despite the advantages of the multiomic approach used, limitations may be imposed by the range of tissues, timepoints and technical features of the assays used. For example, we treated tissues as homogenous entities in an approach that ignores the spectrum of constituent cell types and their differentiated roles that single-cell multiomic studies have begun to explore [73]. Further, suboptimal partitioning of the brain at dissection hampered our ability to assign the role of the hypothalamus separately from the brain stem, cerebellum, and olfactory bulb. Consequently, the variation in whole brain transcriptomes among replicates within timepoint was so large as to prevent meaningful analysis. Finally, we were unable to generate high quality chromatin state data from ovary samples despite repeated attempts. An incomplete compendium of tissues and datatypes has resulted in an imperfect view of the changing epigenomic landscape.

The promise of multiomic data will remain unfulfilled without methodological approaches capable of identifying system perturbations associated with phenotypic change. Here, we used a gene regulatory network approach which has proven successful for investigation of puberty and other complex traits [20, 65, 66]. We identified TRIM25, a gene encoding the estrogen-responsive finger protein, as the network element undergoing the most dramatic change in connectivity and strongly suggests it plays a key role. Looking forwards, it is tempting to speculate that several genes identified represent high value targets for manipulation via gene editing in an attempt to delay or ablate sexual maturation. Among the range of putative targets identified, the core components of the SWI/SNF chromatin remodeling complex (*ARID1B* and *SMARCA2*) are appealing due to their ability to exert wide ranging change in gene expression. The results described may therefore lead to better management of unwanted early maturation within an aquaculture setting where the completion of maturation is associated with reduced product quality and production inefficiencies.

## Materials and methods

### Experimental design

Animals were managed using photoperiod manipulation to synchronize the timing of commitment into maturation. A population of female brood stock were used that were ~ 36 months post fertilization in April 2017. The management of the animals and associated timeline for sampling events is given in Fig. 1A. In order to measure and control for variation between individuals, 4 fish (biological replicates) at each of the four time points (T1-T4) were used. The maturation status of animals (leading up to the long day photoperiod initiation) was monitored by ultrasound. Control samples at T1 time point were collected on mid-June 2017 before induction of maturation occurred late-June 2017. Following the application of the long photoperiod, tissues were sampled at different three time points in 2 weeks intervals (T2-T4). At each sampling event, the gonadosomatic index GSI was calculated from the ovary mass as a proportion of the total body mass as follows:

$$\mathrm{GSI}=\left[\mathrm{ovary}\ \mathrm{weight}/\mathrm{total}\ \mathrm{body}\ \mathrm{weight}\right]\times 100.$$

### RNA isolation, RNA-seq library preparation and sequencing

Tissue samples were preserved in RNA-Later at − 80 °C and total RNA was isolated using RNeasy mini kit (QIAGEN) as previously described [71]. Tissues were lysed twice in 450 μL of lysis solution on a Precellys 24 homogenizer for 30s at 4.0 ms − 1. RNA was washed twice before elution with 40 μL at room temperature. RNA quality was checked using a NanoDrop ND-1000 spectrometer, Qubit 2.0 fluorometer and Agilent 2100 bioanalyzer. Messenger RNA (mRNA) was isolated from 1 μg of total RNA. Sixty-four RNA-Seq libraries (4 time points × 4 tissues × 4 biological replicates) were prepared using the TruSeq RNA Sample Preparation Kit (Illumina). Libraries were sequenced on Illumina Nova-Seq 6000 sequencing platform at the Australian Genome Research Facility (AGRF) in Melbourne, Australia. Sequencing produced a total of 4.4 billion individual 150 bp paired-end reads and ~ 70 million PE reads per library (Data S1).

Transcriptomic data quality control (QC), genome mapping and read counting RNA-seq reads were checked for quality using FastQC software. High quality reads (Q > 30) were mapped to the Atlantic salmon genome ICSASG_v2 [74] using TopHat2 version 2.1.1 [75] using the default parameters. BAM (alignment) files were sorted by read name and converted into SAM format using SAMtools version 1.4 [76]. The package HTSeq version 0.7.2 [77] was applied to count unique reads mapped to exons using default parameters except for “reverse” with the strandedness.

### Genomic DNA isolation, WGBS library preparation and sequencing

Tissue samples were snap frozen in liquid Nitrogen (LN_2_) and stored at − 80 °C until genomic DNA (gDNA) was extracted using DNeasy blood and tissue kit (QIAGEN). Tissues were lysed in 360 μL of lysis solution on a Precellys 24 homogenizer for 30s at 4.0 ms − 1. Samples were incubated with 40 μL of Proteinase K enzyme at 56 °C for 1 h. Following lysis, samples were treated with RNase (8 μL of RNase A incubated for 2 min at room temperature). DNA was bound to the provided columns, washed twice and eluted in 100 μL at room temperature. gDNA purity were assessed by gel electrophoresis and NanoDrop ND-1000 spectrometer. DNA concentration and integrity were assessed using Agilent 2100 bioanalyzer. gDNA was fragmented (200-400 bp) by sonication using Covaris S220, followed by end repair/adenylation and adapter ligation. Bisulfite modification was performed to the DNA fragments using the EZ DNA Methylation-GoldTM Kit (Zymo Research, Inc.). Twelve libraries prepared from 3 tissues (pituitary, ovary and liver), 2 time points (T1 and T4) and 2 biological replicates. Libraries were sequenced on HiSeq 2500 sequencing platform at Novogene, Hong Kong. Sequencing produced a total of 2.2 billion individual 150 bp paired-end reads and 185 M PE reads per library. Bisulfite conversion rates (percentage of C changed to T after bisulfite treatment) were consistently > 99.8%.

### WGBS data QC, genome mapping and methylation calling

Raw data quality control was performed using Trim Galore v0.5 (http://www.bioinformatics.babraham.ac.uk/projects/trim_galore/) to filter bases (Q scores < 30) and remove both universal and indexed adapter sequences. Processed high-quality data were mapped to into a bisulfite-converted version of the Atlantic salmon reference genome ICSASG_v2 [72] using BSseeker2 v2.1.8 [78] with default parameters for aligning paired-end libraries using Bowtie2 [79]. PCR duplicates were removed using Picard MarkDuplicates (http://broadinstitute.github.io/picard/). Filtered (duplicates-free) reads (110 M PE reads) were retained for downstream methylation analysis with an average genome coverage of 11x in pituitary, ovary and liver. Methylation calling was conducted using the Python script call-methylation.py within BSseeker2. CGmap files were used for subsequent exploratory and differential methylation analyses. The mstat command within CGmap tools was used to generate global and CG context (CG, CHG, CHH) DNA methylation levels [80].

### DNA methylome exploratory analyses

As CG methylation contributed to the bulk of methylated Cs, average methylation levels of genome-wide CpG positions were calculated in 50 kb bins across the genome using mbin command within CGmap tools and plotted as Violin plots using the R package vioplot, https://cran.r-project.org/web/packages/vioplot/index.html. To begin assessment of the quality of our libraries, common CpGs with minimum 10x coverage among the 12 samples were used in PCA using prcomp implemented in R. Correlation matrices (based on Pearson coefficient) were prepared using the R package corrplot (https://cran.r-project.org/web/packages/corrplot/index.html). Hierarchical clustering analysis was conducted with hclust implemented in R using compute linkage and Euclidean distances.

### Nuclei extraction, ATAC-seq library preparation and sequencing

ATAC-seq libraries were prepared from snap-frozen tissues using the Omni-ATAC method [81] with few modifications. A mortar and pestle were used to grind frozen tissue (20 mg) in LN_2_ using. The pulverized tissue powder was transferred to a pre-chilled 2 ml dounce homogenizer containing 1 mL cold 1x homogenization buffer and homogenized with the pestle to form a uniform suspension (10–20 strokes). The homogenate was filtered with a 40uM nylon cell strainer (BD Falcon) before layering onto the iodixanol solution as described previously 69. The ratio of nuclei to enzyme concentration was optimised for each sample by performing transposition reactions containing 50,000, 100,000 and 200,000 nuclei with 2.5ul of tagment enzyme in 50ul of transposition mix [81]. The transposed DNA was amplified with custom primers as described elsewhere [82], before libraries were purified using Agencourt AMPure XP beads (Beckman Coulter) and quality controlled using a Bioanalyzer High Sensitivity DNA Analysis kit (Agilent). Twelve liver ATAC-seq libraries arising from 3 biological replicates × 4 time points (T1-T4) were sequenced at the IMB sequencing facility (University of Queensland) on an Illumina NextSeq 150 cycle (2 X 75 bp).

### Chromatin accessibility data QC, genome mapping and peak calling

Sequencing produced a total of 1.2 billion individual paired-end reads (Data S8). Raw reads were mapped to the Atlantic salmon reference genome ICSASG_v2 58 using BOWTIE2 version 2.3.5.1 with the *--very-sensitive* parameter [77]. Duplicate reads were removed using the MarkDuplicates function in Picard tools (version 1.119) http://broadinstitute.github.io/picard/). Multi-mapped reads and mitochondrial reads were filtered out and only uniquely mapped reads (MAPQ > 10) were extracted from alignment files using SAMTOOLS for downstream analyses. For peak calling, the model-based analysis of ChIP-seq (MACS2) (https://github.com/macs3-project/MACS) was used to identify read enrichment regions (peaks) using default parameters “-f BAMPE”. Only peaks detected in at least two replicates per condition were used for downstream analyses, and peaks across timepoints were merged to generate a list of consensus peaks. The number of raw reads mapped to each peak was quantified using the Python package HTSeq version 0.11.1 [75].

### Statistical analyses

#### Differential gene expression and clustering analyses

The edgeR package [83] was used to analyse raw counts within the R statistical computing environment to infer differential gene expression among tissues. The four tissues at the long photoperiod time points (T2, T3 and T4) were compared to the control samples at T1. *P*-values for differential gene expression were adjusted for multiple testing using the Benjamini and Hochberg algorithm [84]. For further analyses of differential expression, only genes with a false discovery rate (FDR) of < 0.05 and have at least absolute log2(fold change) > 1 were considered significant. PCA was conducted on the lists of significant DEGs using normalised expression data (log_2_FPKM) using the function *--prin_comp* within trinity. Hierarchical clustering analysis was conducted using trinity’s utility analyze_diff_expr.pl on significant DEGs in each tissue where mean-centred normalized expression (log_2_-transformed FPKM+ 1) were compared across time points [85]. Gene clusters with similar expression patterns were obtained using the Perl script define_clusters_by_cutting_tree.pl within trinity to cut the hierarchically clustered gene tree into clusters with similar expression using the *--Ptree* option.

### Gene ontology (GO) enrichment of the identified co-expressed gene clusters

To infer the functions of the gene clusters, gene ontology (GO) enrichment was performed to identify the enriched biological themes using the R package clusterProfiler version 3.9 using default settings [86]. The ENTREZ gene identifiers of up- and downregulated clusters per tissue were used as query gene list against the background genes in each tissue. For the enrichment analysis, GO terms with a corrected *P*-value of < 0.05 were considered significant. Categories of candidate genes implicated in maturation were visualized as heatmaps using their normalised expression values with the R package pheatmap version 1.0.12 https://cran.r-project.org/web/packages/pheatmap/pheatmap.pdf.

### Inference of master regulators

Master regulator analysis was performed using regulatory impact factor (RIF) metrics described by [87] to identify key regulators contributing to the transcriptional divergence in the T4 vs T1 comparison per tissue. Predicted transcription factors (TFs) in Atlantic salmon genome were obtained from a previous salmon transcriptomic study [71]. Normalized data of these genes were retrieved for T1 and T4 samples in this current study. As most of the transcriptional changes were detected at T4, RIF was applied only to the T4-T1 comparisons for each tissue. Genes were filtered based on expression, genes with a mean expression FPKM < 0.2 were excluded. Briefly, RIF exploits the differential co-expression concept where regulators were contrasted against unique lists of genes that were differentially expressed at T4 (compared to T1) per tissue. RIF comprises of two different metrics that assign scores to putative regulators consistently differentially co-expressed with target genes (RIF1), and to those able to predict the abundance of target genes (RIF2). Those scores deviating ±2.57 standard deviation from the mean were considered significant (corresponding to a t-test *P* < 0.01). The regulators identified were used as input for construction of gene regulatory networks as summarized in Supplementary Fig. S1b.

### Differential CpG methylation analysis

The R package DSS was used to identify differential methylation regions using common CpGs [88]. In each tissue, two replicates at T4 were compared to the control samples at T1 based on CpG methylation levels. At each CpG site, the methylation (M) level was calculated as a proportion of the total counts (coverage) as follows: M levels = [methylated counts / total counts] × 100. DSS was selected as it considers the biological variation among replicates (characterized by a dispersion parameter) and the sequencing depth. Differentially methylated loci (DMLs) were identified by estimating mean methylation levels for all CpG sites followed by estimating dispersion at each site and conducting a Wald test (*P* < 0.001). Smoothing (combining information from nearby CpG sites to improve the estimation of methylation levels) was utilised to obtain mean methylation estimates in WGBS data where the CpG sites are dense. Based on the DML results, regions with statistically significant CpG sites were identified as a differentially methylated regions (DMRs) with minimum length/distance of 50 bp and minimum CpG coverage of 3. Mean methylation between groups of greater than 10% (delta = 0.1) and *P* < 0.001 was considered significant. A circos plot was produced to visualize multi-tissue genome-wide DMRs using Circos (http://circos.ca/software/). Individual DMRs were also visualized using the *showOneDMR* function within the DSS package to plot both the methylation percentages (including a smoothed curve) as well as the coverage depths at each CpG site.

### DMR annotation, DMGs/DEGs correspondence analysis

Differentially methylated regions were compared against the protein coding gene set annotated on reference ICSASG_v2 using custom Perl scripts. This classified DMRs as overlapping a gene body (genic), 5 kb upstream of a transcription start site TSS (putative promoter), 5 kb downstream of TSS (5 kb downstream), or otherwise intergenic. The distance between each DMR and nearest gene is provided in Data S5, S6. The overlap between significant genes from differential expression and methylation was checked using the intersect function within bedtools [89].

### GO enrichment of DMGs

GO enrichment analyses were conducted on both the sets of hypermethylated genes (*n* = 1156) and genes found to be hypermethylated and upregulated in ovary (*n* = 148) using the R package clusterProfiler. Genes driving GO enrichment were plotted as a heatmap using the R package pheatmap as above.

### Differential accessibility and clustering analyses

Samples from the long photoperiod time points (T2, T3 and T4) were compared to control samples (T1) for each tissue. Raw counts were analyzed using the R package edgeR and *P*-values were corrected for multiple testing using the Benjamini and Hochberg algorithm. Peaks with FDR < 0.05 and log2FC > ± 1 were considered significantly differentially accessible regions (DARs). PCA of significant DARs used normalized accessibility data (log2CPM) prepared using the function --prin_comp within Trinity. Hierarchical clustering analysis was conducted using analyze_diff_expr.pl where mean-centred normalized accessibility (log_2_CPM + 1) were compared across time points [83]. Gene clusters with similar accessibility patterns were obtained using the Perl script “define_clusters_by_cutting_tree.pl” to cut the hierarchically clustered gene tree into clusters with similar accessibility patterns as described above.

### Genomic distribution of DARs within clusters

Hierarchical clustering identified both accessible and inaccessible DAR clusters. DARs per cluster were annotated in a genomic context (genic, promoter, 5 kb downstream or intergenic) as previously done for annotation of DMRs.

### ATAC-seq and RNA-seq correspondence analysis

Only DARs co-located with genes and promoters were used for co-analysis with gene expression data. The relationship between accessibility of DARs and gene expression was visualized by overlying information of significant DARs to genome-wide normalized expression estimates in liver samples and plotted as a MA-biplot. A linear regression analyses were performed to assess correlations between accessibility and expression abundance and the effect of changes in accessibility and changes in gene expression across time. Chromatin accessibility and gene expression data were visualized using Gnuplot version 5.0.7 (http://www.gnuplot.info) by overlying accessibility data of significant DARs at genes and promoters to genome-wide normalized expression estimates at each timepoint.

Multiomic (transcription/accessibility) heatmap analysis per time point and genomic regions.

All heatmaps were produced using the R package pheatmap using normalized gene expression/accessibility values. GO enrichment analyses have been conducted on the set of nearest genes to accessible promoters using the R package clusterProfiler as described above. The integrated genome viewer (IGV) was used to visualize the relationship between accessibility and gene expression in a 15 kb region that contains HMGRC gene and its promoter region.

### Motif enrichment analyses

The function findMotifsGenome.pl within Homer software version 4.11 (http://homer.ucsd.edu/homer/) was used with default parameters to find sequence motifs significantly enriched among accessible DARs vs inaccessible DARs located within promoter regions.TF motifs that are highly enriched (*P* value < 1 × 10^− 10^) were selected.

### Gene regulatory network (GRN) analyses

Genes from different omics analyses (DEGs, DMGs, DMPs, DACs) along with transcription factors identified by RIF (key TFs), as well as information from published work for tissue-specific (TS) genes and gene-harboring GWAS SNPs (SNPs) were selected based on overlap (at least twice) and mean normalized expression (at least 0.2 FPKM) for network construction. The R package UpSetR (https://cran.r-project.org/web/packages/UpSetR/vignettes/basic.usage.html) was used to investigate the overlap among genes from different sources based on presence/absence data.

For gene network inference, the Partial Correlation and Information Theory (PCIT) algorithm was applied to the selected genes [90] to identify significant connections (edges) between genes (nodes), considering all samples for this initial network. PCIT tests the relationships between all possible three-combinations of genes (trios) to determine correlations between gene pairs considering the influence of other genes present within the dataset. Hence, this algorithm determines the significance of the correlation between given gene pairs after taking into account all the other genes included in the network. PCIT has been extensively used for integrating multitissue multiomics data to gain insights into regulatory networks associated with complex traits (Alexandre et al. [20]; Gòdia et al. [21]; Botwright et al. [22]). Connections between gene nodes were considered significant when the partial correlation was greater than 1.96 standard deviations from the mean (corresponds to *P* < 0.05). The output of PCIT was visualized using Cytoscape Version 3.7.2 [91].

To explore differential connectivity during maturation onset, two other networks were created using the same set of genes (used in the initial network): one using 12 samples at T1 (pre-maturation) and a second using 36 samples at T2, T3 and T4 (post-maturation).

The number of connections of per node was computed in both networks to identify differentially connected genes (DCGs) upon the transition to puberty. Based on the degree of differential connectivity the top 20 DCGs were identified. From these networks, we explored a series of subnetworks based on 1) the top trio DCGs and the top regulators (TFs) among based on the degrees of connectivity between pre-and post-maturation.

## Supplementary Information

**Additional file 1.**

**Additional file 2.**

**Additional file 3.**

**Additional file 4.**

**Additional file 5.**

**Additional file 6.**

**Additional file 7.**

**Additional file 8.**

**Additional file 9.**

**Additional file 10.**

## Data Availability

All data, code, and material used in the analyses are available. Sequencing reads, raw and processed data used in this multiomic study have been submitted to the NCBI Gene Expression Omnibus (GEO) database under Accession numbers GSE157003 (combined data types); GSE157001 (RNA-seq); GSE156998 (ATAC-seq) and GSE156997 (whole genome bisulphite sequencing data). All codes used for gene expression (RNA-seq; https://github.com/AminRM/salmon_mat_transcriptomes), DNA methylation (WGBS; https://github.com/AminRM/DNAmethylomes), chromatin accessibility (ATAC-seq; https://github.com/AminRM/Salmon-Chromatin-Accessibility), gene networks (GRN; https://github.com/AminRM/maturation_GRN) analyses are available.
