# Supplementary material for: Leveraging transcriptome and epigenome landscapes to infer regulatory networks during the onset of sexual maturation
Source: BMC Genomics. 2022 Jun 1;23:413. doi: 10.1186/s12864-022-08514-8 (PMC9158274; doi:10.1186/s12864-022-08514-8)
Supplement: Supplementary file 1 — Additional file 1. [file 12864_2022_8514_MOESM1_ESM.docx]

**Supplementary Information for**

**Leveraging transcriptome and epigenome landscapes to infer regulatory networks driving the onset of sexual maturation**

Amin R. Mohamed ^1$,^ Marina Naval-Sanchez^1$,^ Moira Menzies^1^, Bradley Evans^2^, Harry King^3^, Antonio Reverter^1^, James W. Kijas 1*

*Corresponding author

Email: James.Kijas@csiro.au

**This PDF file includes:**

Supplementary Text

Figs. S1 to S18

Data S1 to S9

References (88 to 90)

Supplementary Figure S1. Schematic view of the bioinformatic workflow.

Supplementary Figure S2. Level of agreement among the four biological replicates at the four sampling events (T1, T2, T3 and T4)

Supplementary Figure S3. Multi-tissue genome-wide differences in gene expression during onset of salmon maturation.

Supplementary Figure S4. Expression level and GO enrichment for the pituitary clusters

Supplementary Figure S5. Expression level and GO enrichment for ovary gene clusters.

Supplementary Figure S6. Ovary DEGs implicated in onset of salmon maturation.

Supplementary Figure S7. Expression level and GO enrichment for liver gene clusters.

Supplementary Figure S8. Characterization of multi-tissue DNA methylomes in Atlantic salmon

Supplementary Figure S9. Multi-tissue genome-wide differences in CpG methylation during onset of salmon maturation.

Supplementary Figure S10. Weak correlation between changes in DNA methylation and gene expression in ovary.

Supplementary Figure S11. Evaluation of ATAC-seq libraries using transcription start site enrichment (TSSs).

Supplementary Figure S12 Positive correlation between gene expression and accessibility at genic regions.

Supplementary Figure S13 Positive correlation between gene expression and accessibility at promoters.

Supplementary Figure S14 Changes in Chromatin accessibility in liver during maturation onset.

Supplementary Figure S15 Accessibility at CREs

Supplementary Figure S16 Known motif enrichment results.

Data S1. (separate file)

Description of multi-tissue transcriptome (RNA-seq) data. Sequencing raw data and mapping statistics for all samples at all time points.

Data S2. (separate file)

Differentially expressed genes (DEGs) in pituitary, ovary and liver (FDR < 0.05; |logFC| > 1) at T2, T3, T4 vs T1. Statistics for differential expression along with raw counts are provided for all samples.

Data S3. (separate file)

Statistics associated with Gene ontology (GO) enrichment for each up- and downregulated gene clusters in pituitary, ovary and liver. Enriched GO categories BP, CC, MF were selected using a hypergeometric test at Bonferroni-adjusted P < 0.05.

Data S4. (separate file)

Transcription factors identified as master regulators according to the regulatory impact factor (RIF) metrics in pituitary, ovary and liver tissues (P < 0.01).

Data S5. (separate file)

Description of methylome (WGBS) data, sequencing, mapping and methylation statistics.

Data S6. (separate file)

Methylation statistics, genomic locations and annotation of differentially methylated regions (DMRs) in pituitary, ovary and liver.

Data S7. (separate file)

Statistics associated with Gene ontology (GO) enrichment for hypermethylated genes in ovary and 148 (hypermethylated/upregulated) genes in ovary. Enriched GO categories BP, CC, MF were selected using a hypergeometric test at Bonferroni-adjusted P < 0.05. Expression and methylation levels of biological candidate genes from the 148 hypermethylated/upregulated ovary genes

Data S8. (separate file)

Description of chromatin accessibility (ATAC-seq) data, sequencing, mapping statistics. Differentially accessible regions (DARs) in liver (FDR < 0.05; logFC ±1) at T2, T3, T4 vs T1. Statistics for differential accessibility along with raw counts are provided for liver samples. Genomic locations and annotation of liver differentially accessible regions (DARs) in accessible and inaccessible clusters.

Data S9. (separate file)

Differentially connected genes (DCGs) (10%) between pre- and post-maturation GRNs. The table shows number of connections per gene (connectivity degree) in both pre-and post-maturation, differential connectivity (Post vs Pre), annotation, tissue of maximum expression and attributes for the different categories.

Supplementary Information Text

**Supplementary results.**

**Salmon multi-tissue transcriptome profiling during maturation:**

A total of 4.4 billion 150 bp paired-end (PE) reads were obtained from 64 RNA-Seq libraries (~70 million PE reads per library). Hierarchical clustering of expression data revealed agreement among the biological replicates in each tissue and resolve the long photoperiod treatment (T2, T3 and T4) from the control samples at T1, as shown in the pairwise Spearman correlations between samples except for the brain samples that showed inconsistency among replicates, hence excluded from further analyses (Supplementary Fig.S2). The brain samples originated from heterogenous brain tissue of multiple regions and the sampling regions weren’t consistent throughout the experiment. The brain-pituitary-gonad (BPG) axis is a key regulator of sexual development in vertebrates. Activation of neurons in the hypothalamus leads to production of gonadotropin releasing hormones (GnRH), which stimulate the release of gonadotropins from the pituitary gland that induce the production of gonadal steroids, which in turn affect various aspects of sex-related physiology, secondary sexual characteristics and behaviour. In the next sections, the transcriptome results from the three tissues are investigated.

Pituitary transcriptome profiling revealed few genes (n=10) were differentially expressed, of which 7 and 3 genes were down- or upregulated, respectively in the pituitary gland at T2. However, the response at T3 and T4 involved more genes; 221 DEGs (75 and 146 genes were down- or upregulated) at T3 and 213 DEGs (76 and 236 genes were down- or upregulated) at T4. The range of log2(fold-change) and false discovery rates are summarized (Supplementary Fig.3; Data S1). Upregulated gene cluster showed significant GO enrichment with respect to 23 GO-BP (Biological Process), 13 GO-CC (Cellular Component) and 38 GO-MF (Molecular Function) terms (Supplementary Fig.S4 and Data S5) related to maturation related functions as G protein−coupled receptor signalling and hormone activity. While 32 GO-BP and 10 GO-MF terms related to receptor ligand activity and developmental process involved in later stages of reproduction (derived from the presence of many genes of ZP3 encoding zona pellucida sperm-binding protein 3 that is essential for sperm binding during fertilization) showed significant enrichment among the downregulated gene cluster (Supplementary Fig.S4).

In ovary, significant transcriptome remodelling occurred during maturation that involved differential expression of ~ 6000 genes after maturation induction (ie: at T2, T3 and T4) compared to the control T1 (adjusted P < 0.05) (Supplementary Fig.S3). 466 ovary genes were differentially expressed at T2 (63 and 403 genes were down- or upregulated). At T3, 1790 were differentially expressed (81 and 1709 genes were down- or upregulated). At T4, the ovarian transcriptome underwent extensive remodelling of 3737 genes (240 and 3497 genes were down- or upregulated) (Data S3). Hierarchical clustering of the ovary DEGs revealed distinctive expression profiles for post-maturation and identified two distinct clusters of upregulated genes (n=3476; 58% of ovary DEGs) and downregulated genes (n=301) (Supplementary Fig.S5). Upregulated gene cluster showed significant GO enrichment with respect to 145 GO-BP, 15 GO-CC and 75 GO-MF terms related to cell adhesion, immune/inflammatory response, development (Supplementary Fig. 5c; Supplementary Table 6). While 16 GO-MF terms related to channel activity were enriched among downregulated cluster (Supplementary Fig.S5; Data S6). Ovary DEGs were grouped based on their functional profile into gene categories that are implicated in onset of sexual maturation (Supplementary Fig.S6).

4001 genes were differentially expressed in liver at T2 (1821, 2180 genes were down- and upregulated). At T3, 2153 were differentially expressed (957, 1196 genes were down- and upregulated). At T4, the liver transcriptome underwent extensive remodelling of 3387 genes (1301, 2086 genes were down- and upregulated) (Data S4). Hierarchical clustering of the liver DEGs revealed distinctive expression profiles for post-maturation and identified two distinct clusters of upregulated genes (n=3,336) and downregulated genes (n=2,347) (Supplementary Fig.S7). Upregulated gene cluster showed significant GO enrichment with respect to 45 GO-BP, 17 GO-CC and 23 GO-MF terms related to organic acid metabolic processes and mitochondrial transport (Supplementary Fig. 7 and Supplementary Table 7). While less terms were enriched among downregulated cluster; 14 GO-BP, 13 GO-CC and 8 GO-MF terms related to translation and developmental process involved in reproduction mainly sperm-egg recognition during binding of sperm to zona pellucida (Supplementary Fig.S7 and Data S7).

**Salmon multi-tissue DNA methylome profiling based on WGBS data:**

Methylome sequencing produced 4.4 billion reads, with average coverage of ~18x for each of the 12 DNA methylome libraries (2 replicates x 2 conditions x 3 tissues) (Data S9). We plotted the average CpG methylation levels in genomic bins to show the methylation levels are within the 0 to 1 range (Supplementary Fig.S8). Clustering analysis of the common CpGs in the 12 DNA methylome libraries revealed three main clades that grouped samples according to tissue type, but also separated the samples according to the maturation status. PCA analysis of common CpGs showed high variation among the ovary samples implying extensive remodelling in the ovarian methylomes (Supplementary Fig.S8).

**Chromatin accessibility data (liver):**

We obtained a total of ~750 million individual paired end reads from 12 liver libraries (3 replicates x 4 time points). An average of 93% mappability (duplicates free uniquely mapped reads) and 29 million qualified paired-end reads (sequencing fragments) per library were obtained. On average, ~94,000 high-confidence open chromatin regions (or peaks) were identified across liver and ovary libraries (Data S16). These peaks were enriched around transcription start sites (TSSs) (Supplementary Figure S11).

Differential accessibility analyses revealed a strong early remodelling in the chromatin state landscape, as most DARs were observed at T2 (n=1,501) before decreasing in stepwise fashion at T3 (n=477) and T4 (n=148; Data S17, S18). We next asked if the early changes in chromatin state persisted throughout the time course using hierarchical clustering. The majority of DARs (n=1,036 or 57%) exhibit reduced accessibility at T2 compared with T1 and subsequently remained unchanged at later time points (Supplementary Figure S14). Similarly, regions that gained accessibility at T2 (n=696 or 38%) also remained unchanged at later timepoints. This left less than 10% of DARs (n=99) that displayed an oscillating pattern following the onset of the maturation. Together, this revealed the ATAC-seq signatures were predominantly stable chromatin state changes, as opposed to pulsatile epigenomic changes.

We focused on the subset of CREs that underwent significant increase in accessibility during the time course (Supplementary Fig.S15) to evaluate i) the expression behaviour of their closet gene; ii) the biological function of those genes, and iii) any enrichment for transcription factor binding sites. To provide a fine-scale view of this cis-regulation, a 15 kb region of Ssa15 spanning the CRE and exons of a gene that regulates hepatic lipid metabolism (HMGCR) (88) is provided in Supplementary Fig.S15. The final analysis used HOMER to search for TF motifs that bind master regulators driving gene transcription (89). Specifically, we computed the enrichment of TF motifs in CREs that gained chromatin accessibility against a background that remained inaccessible. This revealed significant enrichment of 13 motifs, corresponding to the preferred binding sites of specific transcription factors present in 29 - 58% of targets following the onset of maturation (Supplementary Figure S16). Among these, the most significant motif matched the global transcriptional regulator E3 Ubiquitin-Protein Ligase CNOT4 that regulates essentially every aspect of gene expression, from mRNA synthesis to protein destruction including the degradation of RNAPII (90). The results strongly suggest that chromatin state changes at CREs directly control gene expression in liver and upregulate energy metabolism genes via changes in TF activity.

**Supplementary Figure S1.** Schematic view of the bioinformatic workflow for **a** Analyses of RNA-seq, WGBS and ATAC-seq data. Illumina reads were mapped onto the Atlantic salmon genome and alignment files were processed to extract gene expression counts, CpG methylation, peak counts for downstream analyses of differential analyses using different packages in the R environment. **b** Inference of master regulators and gene regulatory network (GRN) construction. RIF metrics were used to identify master regulators contributing to differential expression observed at T4 in pituitary, ovary and liver. The unique list of these regulators was integrated with other multi-omics results that included differentially expressed genes from three tissues (DEGs), differentially methylated genes and promoters from three tissues. Differentially accessible chromatin (co-located with gene bodies and promoters regions) from liver. Along with data about genes-harboring SNPs and tissue-specific (TS) genes obtained from previous maturation GWASs and tissue-specific transcriptomes derived from the same samples. Gene networks were used to integrate the previous results the PCIT algorithm.


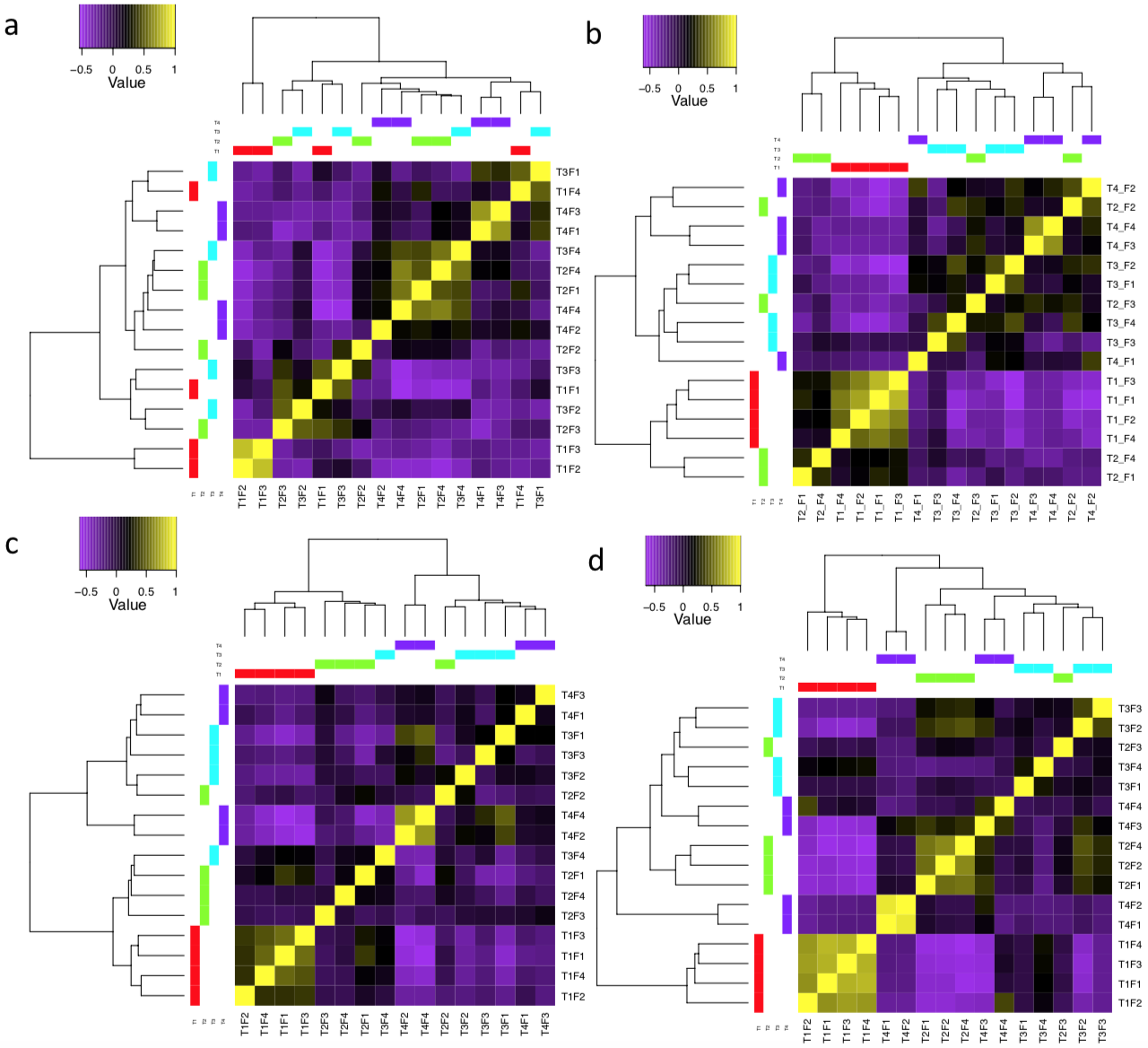


**Supplementary Figure S2.** **Level of agreement among the four biological replicates at the four sampling events (T1, T2, T3 and T4).** The heatmaps show the hierarchical clustered Spearman correlation resulting from comparing genome-wide expression values (log_2_CPM) for all samples against each other in brain **a**, pituitary **b**, ovary **c**, and liver **d**. The level of correlation is presented by a colour field. Sample clustering reveal grouping of the samples from mature fish specially at T3 and T4 in pituitary, ovary and liver. Brain samples failed to achieve such level of grouping due to inconsistent sampling of the heterogenous brain tissue, hence the brain samples were excluded from further analyses.

**Supplementary Figure S3. Multi-tissue genome-wide differences in gene expression during onset of salmon maturation**. An adjusted *P* < 0.05 and logFC > ±1 was used to identify significant differentially expressed genes (DEGs). The overlap among down- and upregulated genes along with hierarchical clustering are shown for **a** pituitary, **b** ovary and **c** liver. It’s worth noting that *GLHA2* (the common subunit present in gonadotropins) was consistently upregulated in the pituitary throughout the experiment. The clustering shown was obtained by comparing normalised expression (FPKM) for samples at T2, T3 and T4 compared to the control at T1. Expression values were log_2_-transformed and mean centred by gene. The relative expression values are shown in yellow-navy scale.

**Supplementary Figure S4. Expression level and GO enrichment for the pituitary clusters. a**, **c** Trends in gene expression for the up- and downregulated cluster in pituitary. The Y-axis represents the mean-centred log_2_(FPKM+1) value, expression of single genes is plotted in grey while the mean expression of the genes is plotted in blue. **b**, **d** Enriched gene ontology (GO) terms (hypergeometric test, Bonferroni-adjusted P < 0.05) among the list of the up- and (N=333) downregulated (N=125) and the gene ratio for the genes that map to each term.

**Supplementary Figure S5. Expression level and GO enrichment for ovary gene clusters**. **a, b** Trends in gene expression for the up- and downregulated clusters in ovary. The Y-axis represents the mean-centred log_2_(FPKM+1) value, expression of single genes is plotted in grey while the mean expression of the genes is plotted in blue. **c**, **d** Enriched gene ontology (GO) terms for each cluster (hypergeometric test, Bonferroni-adjusted P < 0.05 and the gene ratio for the genes that map to each term.

**Supplementary Figure S6. Ovary DEGs implicated in onset of salmon maturation**. The multi-panel heat maps show genes implicated in and/ or encoding **a**, steroidogenesis and hormone receptors. **b**, growth factors. **c**, extracellular matrix remodeling. **d**, follicular development. **e**, immune- and inflammatory responses. The clustering shown was obtained by comparing normalized expression (FPKM) for samples at T2, T3 and T4 compared to the control at T1. Expression values were log_2_-transformed and mean centered by gene. The relative expression values are shown in yellow-navy scale.

**Supplementary Figure S7. Expression level and GO enrichment for liver gene clusters**. **a, b** Trends in gene expression for the up- and downregulated clusters in liver. The Y-axis represents the mean-centered log_2_(FPKM+1) value, expression of single genes is plotted in grey while the mean expression of the genes is plotted in blue. **c**, **d** Enriched gene ontology (GO) terms for each cluster (hypergeometric test, Bonferroni-adjusted P < 0.05 and the gene ratio for the genes that map to each term.

**Supplementary Figure S8. Characterization of multi-tissue DNA methylomes in Atlantic salmon**. **a**, Global DNA methylation levels at different CG context (CG, CHG and CHH) in liver (L), ovary (O) and pituitary (P) at the two replicates of the control (C) and mature (M) samples. CpG methylation contributes to 99.5% of methylated Cs. **b**, Violin plot showing average CpG methylation levels in genomics bins across the twelve methylome libraries shows similar profiles for samples of each tissue. **c**, Correlation matrix of replicates using common CpGs, replicates of the same tissue shows higher correlation and clustered together. **d**, Principal Component Analysis (PCA) using common CpGs separates samples by tissues along PC1 (explaining 29.8% of the variance) and shows extensive variation among ovary methylomes along PC2.

**Supplementary Figure S9. Multi-tissue genome-wide differences in CpG methylation during onset of salmon maturation**. **a**, Hierarchical clustering of the common CpGs in the 12 methylome libraries reveals clustering by tissue type and grouping pituitary and ovary in a cluster and liver in the other. This shows variation in methylation is much lower between replicates of the same sample compared with variation among tissues. **b**, Bar graph showing significant DMRs identified for each tissue and ovary having the highest number of DMRs. A *P* threshold of 0.001 and delta (difference of the two group means) > 10% were used to identify significant differentially methylated regions (DMRs). **c**, Circos plot shows significant differentially methylated regions (DMRs) detected in T4 samples compared to the control T1 in pituitary, ovary and liver (from the outer circle inwards). **d**, Venn diagram shows minimum overlap among DMRs in the three tissues. **e,** Relative distribution of DMRs across genic, promoters, 5kb downstream and intergenic regions of the salmon genome in pituitary, ovary and liver are shown. DMR genomic distribution reveals on average 70% of DMRs were co-located within 5kb of a gene.

 **c**

**Supplementary Figure S10.** Weak correlation between changes in DNA methylation and gene expression in ovary. The MA biplots showing lack of correlation between genome-wide differential gene expression in T4-T1 ovary samples in yellow and methylation levelse in blue-red spectrum reflecting hypo-to hypermethylation at both gene bodies (**a**) and promotor regions (**b**). Linear regression analyses further confirmed very low R-squared values in both cases. **c**, Overlap between sets of DMGs and DEGs detected at T4 vs T1 per tissue.

**Supplementary Figure S11**. Evaluation of ATAC-seq libraries using transcription start site enrichment (TSSs). To check that accessibility data, TSS enrichment was used for signal to noise calculation. The plots show density plots and heatmaps for reads ±1 kb around TSSs for 12 ATAC-seq liver libraries (4 time points x 3 replicates).

**Supplementary Figure S12** **Positive correlation between gene expression and accessibility at genic regions.** 9 MA biplots showing dynamics of genome-wide gene expression and overlain differentially accessible regions at gene bodies in liver at T2, T3 and T4 compared to T1. Chromatin accessibility levels are shown in red-blue spectrum reflecting open-to closed chromatin at gene bodies and the corresponding gene expression in grey colour. Note that R^2^ values are the highest when using gene expression and accessibility from the same time point.


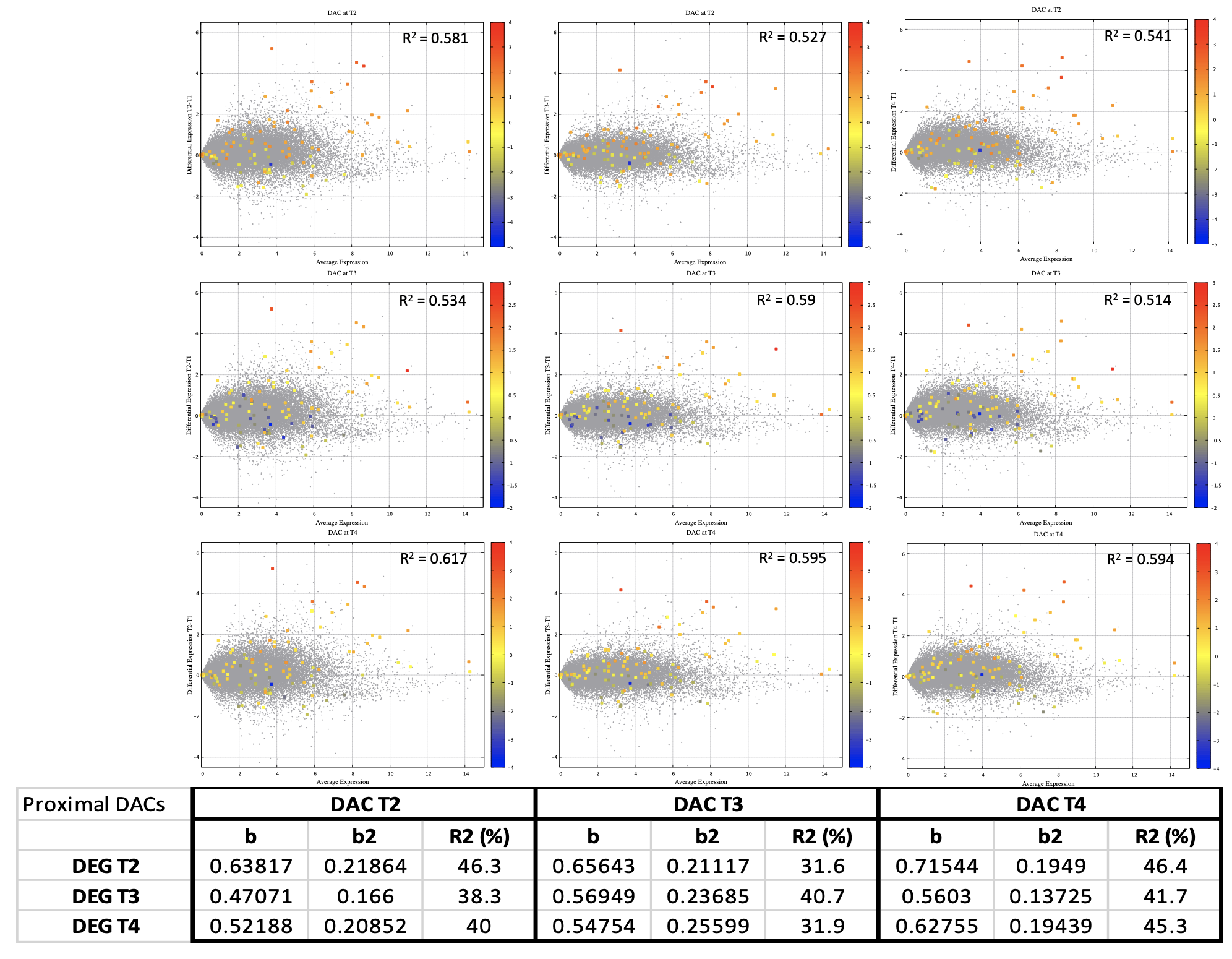


**Supplementary Figure S13 Positive correlation between gene expression and accessibility at promoters.** The 9 MA biplots show genome-wide gene expression and overlain accessibility data for DARs at promoters in liver at T2, T3, T4 compared to T1. The table shows results of a regression analyses conducted on significant DARs located 5 kb upstream of transcription start sites (TSSs) “putative promoters” and gene expression data of the nearest genes.


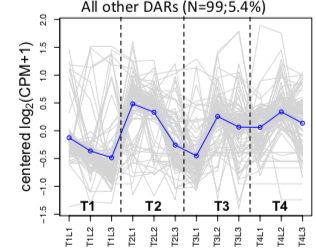

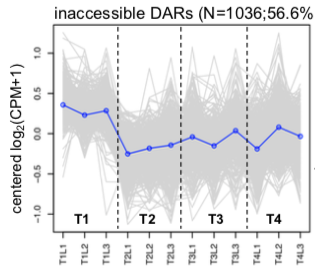

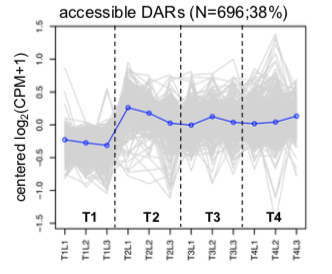


**C**

**Supplementary Figure S14 Changes in Chromatin accessibility in liver during maturation onset. a**, Circos plot shows the genomic distribution of the differentially accessible regions (DARs) (FDR < 0.05 and log2FC > ±1) detected in the triplicates of T2, T3, T4 samples (from outer circle inwards) compared to the control T1. **b**, Hierarchical clustering of unique 1,831 DARs detected at all time points in liver. The clustering shown was obtained by comparing normalised accessibility (CPM data). for samples at T2, T3 and T4 compared to the control at T1. Accessibility values were log_2_-transformed and mean centred by peak. The relative accessibility values are shown in yellow-navy scale. **c**, Co-accessibility analysis reveals stable chromatin states following the onset of the maturation. The majority of DARs exhibited either reduced (n=1036 or 57%) or increased (n=696 or 38%) accessibility at T2 and remained unchanged at later timepoints. Less than 10% of DARs (n=99) displayed an oscillating pattern. The y-axis in each graph represents the mean-centred log_2_(CPM+1) value across time points on the x-axis. Accessibility of single DAR is plotted in grey, while the mean accessibility of each cluster is plotted in blue.

**Supplementary Figure S15 a**, Heatmaps 65 DARs located 5kb upstream of TSSs representing cis-regulatory elements (CREs) that exhibit increased accessibility at T2 along with gene expression of their target (nearest) genes. This showed upregulation of the majority of the associated genes in a tightly coordinated manner (n = 46; 79%, Χ^2^ *p* < 8.028^-08^). **b**, Enriched gene ontology (GO) terms (hypergeometric test, Bonferroni-adjusted *P* < 0.05) among the list of the CREs-regulated genes. This list includes genes involved in hepatic lipid metabolism (*HMGCR*) and energy metabolism (elovl5b and elovl6). **c**, IGV visualisation of a 15 kb region of Ssa15 spanning the CRE and exons of the *HMGCR* gene provides fine-scale view of the coordinated gene expression response to increased accessibility.


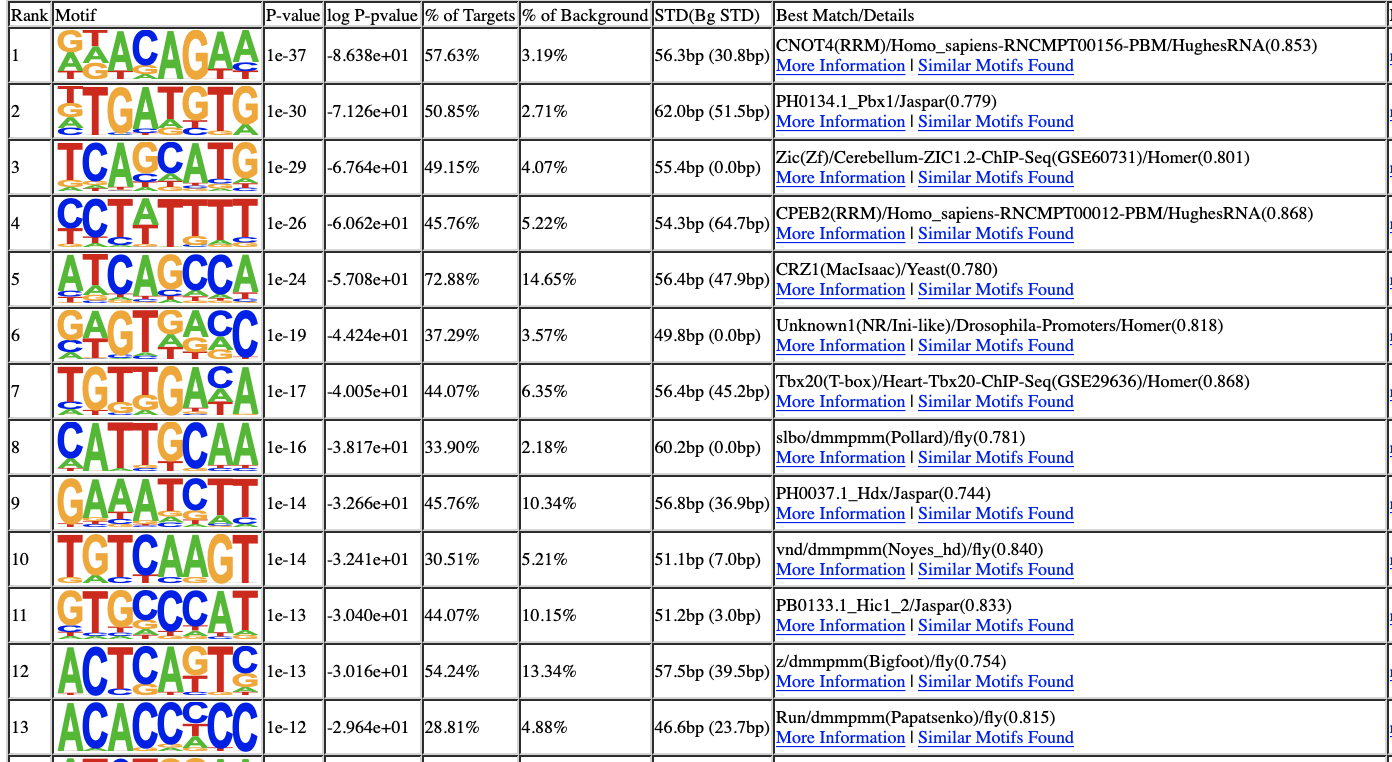


**Supplementary Figure S16** Known motif enrichment results obtained using Homer (*P* < 1e^-10^; at least 25% of targets) by comparing differentially accessible regions at promoters among the accessible cluster to a background of inaccessible regions at promoters. CNOT4 (a E3 Ubiquitin ligase) and Zic (a zinc finger protein) were the matched TFs for the most significantly enriched motifs. This can confirm the importance of zinc finger proteins as key regulators of maturation.


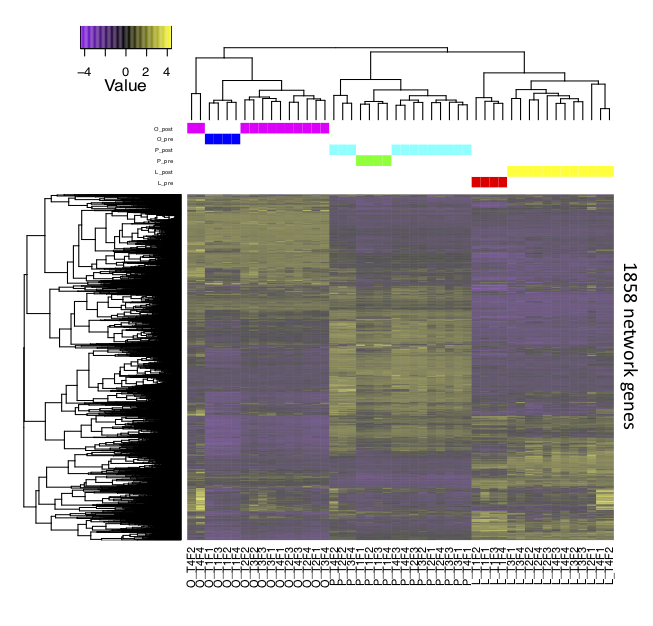


**Supplementary Figure S17.** Heatmap of 1,858 network genes. Genes (rows) and samples (columns) are organized by hierarchical clustering based on Euclidean distances. Samples were clustered by tissue type and the maturation status “pre” (pre-maturation at T1) was resolved from “post” (post-maturation at T2, T3, T4).

**Supplementary Figure S18. Gene regulatory networks constructed using the PCIT algorithm for the pre- and post-maturation samples**. **a**, Networks of the most significant 10% of correlations their respective genes were considered. **b**, Network statistics show increases in the connections in the pituitary that constituted 45% of the post-maturation network connections. All nodes are represented by ellipses except for genes coding key regulators (TFs) have diamond shape. Nodes with yellow borders are differentially methylated, whereas nodes with white labels are differentially accessible between pre- and post-maturation samples. Node colours are relative to the tissue of maximum expression (blue for the pituitary, red for ovary and green for liver). The size of the nodes is relative to the normalized mean expression values in all samples.

Supplementary Figure S19. TRIM25-associated network principal components analysis (PCA). The normalised expression (log2FPKM) values of these genes were used to perform this analysis. PCA grouped samples by first by tissue type, but clearly resolved the pre- and post-maturation status across PC1 that explained 85.1% of the variance.

**SI References**

88. De Jesus DF, Orime K, Kaminska D, Kimura T, Basile G, Wang CH, Haertle L, Riemens R, Brown NK, Hu J, Männistö V, Silva AM, Dirice E, Tseng YH, Haaf T, Pihlajamäki J, Kulkarni RN. Parental metabolic syndrome epigenetically reprograms offspring hepatic lipid metabolism in mice. J. Clin. Invest. 130(5), 2391-2407 (2020)

89. Marnetto, D., Mantica F, Molineris I, Grassi E, Pesando I, Provero P. Evolutionary rewiring of human regulatory networks by waves of genome xpansion. Am. J. Hum. Genet. 102, 207–218 (2018).

90. Jiang, H., Wolgast, M., Beebe, L., Reese, J. Ccr4–Not maintains genomic integrity by controlling the ubiquitylation and degradation of arrested RNAPII. Genes Dev. 33, 705–717 (2019).
